# HierX: Fast multiscale distance-decay interaction on million-node networks

**DOI:** 10.1093/pnasnexus/pgag317

**Published:** 2026-09-17

**Authors:** Alexander Hellervik, Joakim Bohlin, Claes Andersson

**Affiliations:** Department of Physical Resource Theory, Chalmers University of Technology, SE-412 96 Gothenburg, Sweden; Department of Physics, Chalmers University of Technology, SE-412 96 Gothenburg, Sweden; Department of Physical Resource Theory, Chalmers University of Technology, SE-412 96 Gothenburg, Sweden

**Keywords:** hierarchical methods, spatial interaction, network accessibility, sparse operators, distance-decay kernels

## Abstract

Many models across the sciences require global distance-decay interactions on large sparse networks. Gravity models, accessibility measures, spatial economic models, and network influence processes all evaluate distance-weighted potential fields: each location accumulates contributions from every other location, weighted by a decaying function of the shortest-path travel cost between them. Computed directly, such aggregate fields require the dense matrix of all pairwise network costs, which scales quadratically in time and memory. Common approximations either discard long-range contributions or fail when interaction is governed by network distances rather than geometric proximity. We introduce HierX, a hierarchical sparse-plus-correction operator for distance-decay potential fields on networks. HierX constructs multiscale representative layers with explicit correction terms ensuring each location pair contributes exactly once at the finest available resolution. Under bounded-growth assumptions common in spatially embedded networks, applying HierX scales as O(nlogn). Systematic benchmarks confirm quasi-linear scaling to 100,000 nodes. In head-to-head comparison with distance cutoff truncation and Nyström low-rank approximation on 25,000-zone networks, HierX achieves 5–9% root-mean-square error (RMSE) at a fraction of the computational work; Nyström degrades severely on steep decay kernels. Case studies compute population-weighted accessibility on the 2.58-million-node Great Britain driving network and the 1.77-million-node London pedestrian network: a one-time hierarchy construction (∼1 h) yields a compact reusable operator that then evaluates each national-scale accessibility field in under 1 s (∼700 ms for Great Britain, ∼150 ms for London). Open-source code and worked examples are provided.

Significance statement Computing global distance-decay interactions on large networks is a bottleneck across many disciplines: the cost scales quadratically with network size, limiting models to networks far smaller than real-world systems. We introduce HierX, a hierarchical operator that reduces this cost to quasilinear, resolving nearby interactions exactly while approximating distant ones through multiscale aggregation. From Great Britain’s 2.58-million-node road network, HierX builds a compact multiscale interaction operator in about an hour; once stored, the operator evaluates national-scale accessibility surfaces in under 1 s per scenario. This enables applications beyond the reach of quadratic methods: accessibility analysis for transport and land-use planning, gravity-model evaluation, and catchment studies, where many scenarios reuse one network. An open-source implementation makes these capabilities accessible.

## Introduction

Across many domains—transportation, spatial economics, ecology, epidemiology, and network science—models of large-scale systems share a common structure: every node interacts with every other, but the strength of interaction decays with distance (1, 2). Urban accessibility (3, 4), gravity-type spatial interaction models (5, 6), and spatial-economic models (7) all depend on dense matrices of pairwise interactions defined over network-based costs. We focus on transportation and spatial interaction as motivating applications, but the formulation applies to any distance-decay kernel on a sparse graph.

In all these cases, the underlying interaction structure can be represented uniformly as:


(1) $$h_{i}=\sum_{j}f(c_{ij})x_{j},$$

where $c_{ij}$ is a network-based shortest-path cost, *f* is a distance-decay function, $x_{j}$ is an attribute such as population, activity, or neural state, and the output $h_{i}$ is the resulting aggregate potential at node *i*—in transport applications, the accessibility of location *i*.

Equation (1) is a recurring computational kernel within these models rather than a complete representation of them. Gravity and accessibility models may be singly or doubly constrained, so that the interaction between *i* and *j* depends on competition among all destinations, and applications such as congested network loading require explicit paths rather than aggregate potentials. We target the unconstrained kernel (1), which appears repeatedly as a building block—for example, inside the balancing iterations of constrained models—and whose evaluation dominates the cost at scale.

The main obstacle is scale. Computing and storing the full interaction matrix $f(c_{ij})$ requires $O(n^{2})$ time and memory in the number of nodes *n*. For realistic networks of $10^{4}$ to $10^{6}$ nodes, direct computation remains possible but is practically limited to supercomputing resources (terabytes of memory at $n=10^{6}$), placing it out of reach for routine modeling work. Common alternatives each have well-known trade-offs: truncating interactions beyond a distance cutoff is simple and efficient but discards long-range interaction mass—especially problematic for slowly decaying kernels; low-rank and random-feature approximations (8, 9) are effective for positive-definite kernels in Euclidean settings but lack guarantees for non-positive semidefinite (PSD) shortest-path kernels on irregular graphs; local kernels (*k*-nearest neighbors or adjacency-based neighborhoods) preserve short-range structure but sacrifice global reach. These methods can be excellent choices in their respective regimes, but none simultaneously provides subquadratic computation, global coverage, and accurate short-range interactions for network-based distance-decay kernels.

We address this gap with a hierarchical interaction operator that replaces the dense matrix with a multiresolution decomposition, achieving O(nlogn) storage and computation under standard sparsity and bounded-growth assumptions (Complexity analysis section).

### Intuitive overview

The hierarchical operator replaces the exhaustive $n^{2}$ pairwise computation with a multiresolution strategy: nearby locations are treated individually with exact interactions, while distant locations are grouped and interact through representatives. The network is organized into increasingly coarse layers; short-range interactions, which contribute most and are most sensitive to local structure, are resolved exactly, while long-range interactions are approximated through group representatives at a coarseness proportional to distance. Correction matrices prevent double-counting across overlapping layers, ensuring that each pair contributes once at the finest available resolution.

### Contributions

This article presents an operator-level formulation that adapts hierarchical interaction ideas to shortest-path decay kernels on general sparse graphs. We integrate ideas from fast multipole and hierarchical matrix methods, multiscale spatial representations, and adaptive zoning into a unified sparse-plus-correction operator. Our main contributions are:

1. A domain-tailored hierarchical operator for interaction kernels of the form (1) on graph-based shortest-path costs, expressed as
  (2) $$H=\sum_{k}G_{k}^{⊤}(F_{k}-{\mathrm{Corr}}_{k})G_{k},$$
  where $G_{k}$ maps nodes to representatives at layer *k*, $F_{k}$ is the sparse interaction matrix at that layer, and ${\mathrm{Corr}}_{k}$ subtracts coarser-layer contributions to ensure each node pair is counted exactly once.
2. A practical construction of a multilayer hierarchy on graphs, including representative selection, grouping, and sparse storage, designed for geographically embedded networks.
3. A complexity analysis showing that, under standard sparsity and growth assumptions, application scales as O(nlogn) and local network changes can be propagated through the hierarchy with work proportional to the number of affected pairs times the hierarchy depth.
4. An applied and reproducible formulation, with open-source code and worked examples aimed at lowering the barrier to hierarchical interaction methods.

Unlike hierarchical matrix and fast multipole frameworks, which rely on geometric admissibility and low-rank block compression in Euclidean spaces, our operator acts directly on shortest-path metrics of arbitrary sparse graphs, targeting large-scale spatial interaction computation without requiring geometric embedding or positive-definiteness.

### Scope and intended niche

The operator targets the regime where (i) the network is large enough ($n≳10^{4}$) that the quadratic cost of dense computation becomes prohibitive, (ii) the decay function is shallow enough that distant interactions carry meaningful mass, (iii) the application requires repeated evaluations of Hx for varying x, amortizing the build cost, and (iv) the graph is a sparse, roughly spatial network with non-negative edge costs.

### Article outline

The remainder of the article proceeds as follows. *Background and lineage* section reviews the hierarchical lineage from cellular automata (CA) and adaptive zoning, and situates our approach relative to existing hierarchical and graph-based methods. *Methods: the hierarchical operator* section then introduces the hierarchical operator itself, including the construction of representative layers and the sparse-plus-correction formulation. Particular attention is paid to the algorithmic choices that make the method practical on large transport-like networks, including representative selection and grouping. *Complexity analysis–Computational experiments* sections analyze computational complexity, approximation error, and empirical behavior in realistic network settings, including head-to-head comparisons with distance cutoff truncation and Nyström approximation. *Discussion* section discusses limitations, the method’s comparative niche, and directions for future work.

## Background and lineage

### Hierarchical cell-space and the 2002 CA model

This work builds on research originating in CA and complex systems. Andersson et al. (10) introduced a CA/Markov random field model for urban settlement dynamics in which each cell is influenced by the entire lattice, with interaction strengths decaying with distance. A key motivation was what might be called the “speed of light” problem in geographical CA (cf. (10)): finite neighborhoods artificially couple spatial and temporal scales.

To overcome this, the 2002 model introduced a hierarchical cell-space/mean-field renormalization scheme in which the lattice is recursively aggregated into larger blocks carrying average states. Interactions at larger distances are computed between aggregated blocks, reducing the cost from $O(N^{2})$ to approximately O(NlogN) while retaining fine-scale detail locally and providing a multiscale representation of space in which distant influences are represented more coarsely than local ones.

### Variable-grid CA and adaptive zoning

Several authors subsequently generalized the hierarchical cell-space idea. White (11) recast it as a variable-grid CA where distant rings are represented by larger “super-cells”; Van Vliet et al. (12) implemented this for urban growth modeling. Outside the CA framework, Hagen-Zanker and Jin (13) proposed adaptive zoning for gravity-type spatial interaction models, aggregating distant destination zones into larger regions. They note the approach could extend beyond Euclidean distance but do not formulate a general graph-theoretic operator.

Variable-grid CA and adaptive zoning are thus spatial siblings of the present work: they express the idea that spatial detail can be coarser at longer distances but remain tied to raster grids or zonal systems.

### Connections to existing hierarchical and graph-based methods

We briefly describe how the proposed operator relates to existing hierarchical and multiscale methods.

#### Hierarchical matrices and fast multipole methods

The Barnes–Hut treecode (14) and fast multipole methods (15) (FMM) approximate far-field interactions via hierarchical grouping and series expansions, while H-matrices (16–18) compress well-separated blocks in low rank; together these reduce *N*-body interactions from $O(N^{2})$ to O(NlogN) or O(N). Our sparse-plus-correction form (2) shares this near/far decomposition, but there are important differences:

- We work with shortest-path distances on general graphs, not with kernels defined on geometric point sets in Euclidean space.
- We approximate far-field interactions via group representatives and precomputed costs, rather than via low-rank factorizations of block submatrices.
- We focus on domain-specific interaction kernels common in transport and spatial interaction modeling, rather than on general elliptic partial differential equation operators.
- We emphasize dynamic updates to the underlying graph (adding nodes, links, or changing costs), which are less central in classical H-matrix and FMM settings.

Our work is thus an applied, graph-oriented sibling of these methods rather than a competitor to general H-matrix or FMM frameworks. The structural parallel is closest to H-matrices, which treat admissible blocks independently. The $H^{2}$-matrix refinement (18) achieves optimal complexity by expressing cluster bases hierarchically (nested bases), and FMM analogously translates expansions between levels of its tree. Our representative layers are in fact already nested in the set-theoretic sense—the propagation step of Representative selection and grouping section guarantees that each fine-layer group lies within a coarse-layer group—but the operator does not yet exploit this nestedness algebraically: each layer stores its representative costs independently. Two $H^{2}$-inspired refinements appear promising and are discussed as future work in Discussion section: translating cost information between nested layers rather than recomputing it, and replacing the hard single-representative assignment with a weighted average (convex combination) of several representatives, analogous to interpolation-based and variable-order $H^{2}$ constructions. Conversely, the correction-matrix mechanism—which lets overlapping scales coexist while guaranteeing exactly-once contribution without geometric admissibility criteria—may be of interest in continuous settings.

#### Graph Laplacians, spectral sparsification, and diffusion kernels

Spectral sparsification (19) approximates a graph by a sparse subgraph preserving Laplacian quadratic forms. Separately, graph diffusion kernels (20) define interactions via the matrix exponential of the negative Laplacian. Both address a different problem from ours: our focus is on interaction sums $h_{i}=\sum_{j}f(c_{ij})x_{j}$ where *f* is a distance-decay function of shortest-path costs, not a function of the graph Laplacian.

#### Distance oracles, hierarchical routing, and hub labeling

Distance oracles (21), contraction hierarchies (22), transit node routing (23), and hub labeling (24) (see Ref. (25) for a survey) precompute data structures for fast single-pair shortest-path queries. Our objective is different: we compute, for all nodes *i*, a global interaction sum $h_{i}=\sum_{j}f(c_{ij})x_{j}$, which is closer to applying a full operator than answering individual queries. The two families scale differently—contraction hierarchies excel at one-to-one queries, whereas Dijkstra’s algorithm (26) is naturally one-to-many—which suggests a complementary division of labor within our hierarchy: fine layers require many short-range one-to-many searches, for which cutoff-limited Dijkstra is well suited, while coarse layers require costs between relatively few, widely separated representative pairs, exactly the regime where contraction-hierarchy or hub-label queries excel. Such structures could thus serve as a backend for computing the coarse-layer entries of $F_{k}$; we do not pursue this connection here.

#### Graph coarsening and multiscale graph representations

Hierarchical graph pooling methods such as DiffPool (27) learn to map nodes to progressively coarser clusters for graph classification and related tasks. Our hierarchy superficially resembles coarsening, but we do not operate on a reduced graph: instead, we approximate a specific interaction operator and always map back to the full node set. Techniques from graph coarsening could nonetheless inform alternative representative selection strategies.

#### Kernel approximation methods

Random Fourier features (8) and Nyström approximation (9) provide O(n) or O(nlogn) kernel matrix approximations with theoretical guarantees. However, Nyström error bounds rely on the kernel being PSD. Interaction kernels defined on shortest-path costs over general graphs need not be PSD, and in our baseline comparisons (Comparison with alternative methods section) standard Nyström applied to such kernels fails on steep decay functions, consistent with this limitation. Our approach targets a different regime: arbitrary distance-decay functions on graph metrics, where the graph structure itself dictates the hierarchy.

### From spatial hierarchies to general network operators

Existing work does not generalize the hierarchical idea to arbitrary graph structures; does not define a clean algebraic operator; and does not address dynamic structural updates. The present method lifts the hierarchical cell-space idea from regular grids to general sparse networks with non-negative edge costs, defines a linear operator in sparse-plus-correction form, and provides a conceptual framework for dynamic updates with O(logn) propagation depth.

## Methods: the hierarchical operator

### Problem setting

We consider an undirected weighted graph G=(V,E,w) with |V|=n nodes and non-negative edge costs. A designated subset Z⊆V of *zones* carries the activity and accessibility values of interest; the remaining nodes serve only as routing intermediaries. We write $|Z|=n_{z}$. Let $c_{ij}$ be the shortest-path cost between zones *i* and *j* (computed over the full graph), and *f* a distance-decay function. The dense interaction matrix is $F_{ij}=f(c_{ij})$, i,j∈Z. Given an activity vector x, the dense accessibility is


(3) $$h_{i}=\sum_{\;j\in Z}F_{ij}x_{j}.$$

Our aim is to approximate *F* with a hierarchical operator *H* that is much cheaper to evaluate while retaining exact contributions for zone pairs whose costs are known at fine resolution. In the special case Z=V, every node is a zone; all benchmarks in this article use this setting, which represents the worst case for the operator.

### Hierarchical layers and representatives

We build *K* layers. Throughout, shortest-path costs are computed with Dijkstra’s algorithm (26), the classical method that explores a network outward from a source node in order of increasing cumulative cost; it naturally supports one-to-many searches that stop once a cost cutoff is reached. At layer *k*, radius $\rho_{k}$ defines the grouping scale, and the overlap factor α>1 extends the cutoff to $\alpha \cdot \rho_{k}$, controlling how far each layer’s Dijkstra searches reach and thus the resolution of the approximation. Representatives $R_{k}$ are chosen so that each node belongs to exactly one representative at layer *k*. $G_{k}$ is the group-membership matrix.

At the finest layer, $R_{0}=V$. At coarser layers, $\left|R_{k}\right|$ becomes progressively smaller.

### Representative selection and grouping

Representatives and groups are constructed by greedy clustering, layer by layer from fine to coarse, given a base radius $\rho_{0}$, growth factor γ>1 ($\rho_{k+1}=\gamma \cdot \rho_{k}$), and overlap factor α>1 (cutoff $\alpha \cdot \rho_{k}$).

At layer k=0, $R_{0}=V$ and each node represents itself. For k≥1, the candidates are $R_{k-1}$ and the algorithm proceeds:

1. Set $\rho_{k}=\rho_{0}\cdot \gamma^{k}$ and cutoff $\alpha \cdot \rho_{k}$. Initialize $R_{k}=\emptyset$.
2. For each candidate $u\in R_{k-1}$ (ordered by index):
  - If some $r\in R_{k}$ satisfies $c_{ur}<\rho_{k}/2$, assign *u* to the nearest such *r*.
  - Otherwise, promote *u* to $R_{k}$ and reassign any previously grouped candidate *v* for which $c_{vu}<c_{v,r_{k}(v)}$ (takeover).
3. Every member of $R_{k-1}$ now belongs to exactly one group at layer *k*.

The takeover step keeps groups compact by letting a new representative steal nearby nodes from other groups. Because candidates are drawn from $R_{k-1}$, $\left|R_{k}\right|$ shrinks rapidly with *k*.

#### Group propagation and nesting

A coarse-to-fine propagation step ensures every node has a representative at every layer: if node *i* maps to *r* at layer *k* and *r* maps to $r^{\prime }$ at layer k+1, then *i* maps to $r^{\prime }$ at layer k+1. This guarantees *nested* groups—each fine-layer group lies entirely within a coarse-layer group—which the correction matrices require. Group membership is recorded in $G_{k}[r,i]=1$.

#### Cost computation

For each layer *k*, we run cutoff-limited Dijkstra searches from each representative $r\in R_{k}$, storing costs from *r* only to other representatives $u\in R_{k}$ reached within $\alpha \cdot \rho_{k}$. These costs populate the sparse structure $C_{k}$. Applying the distance-decay function *f* to $C_{k}$ yields the interaction structure $F_{k}$ at layer *k*. For the coarsest layer, all representative pairs are stored (no cutoff).

In practice, this is efficient because $\left|R_{k}\right|$ shrinks rapidly with *k*, the cutoff $\alpha \cdot \rho_{k}$ limits each Dijkstra search, and in spatial networks the node count within $\alpha \cdot \rho_{k}$ remains modest. We adopt this greedy algorithm for its transparency and spatial intuition; more sophisticated coarsening schemes could be substituted. Figure 1 illustrates the resulting layers, groups, and stored representative pairs on a 40-zone network.

**Figure 1:**
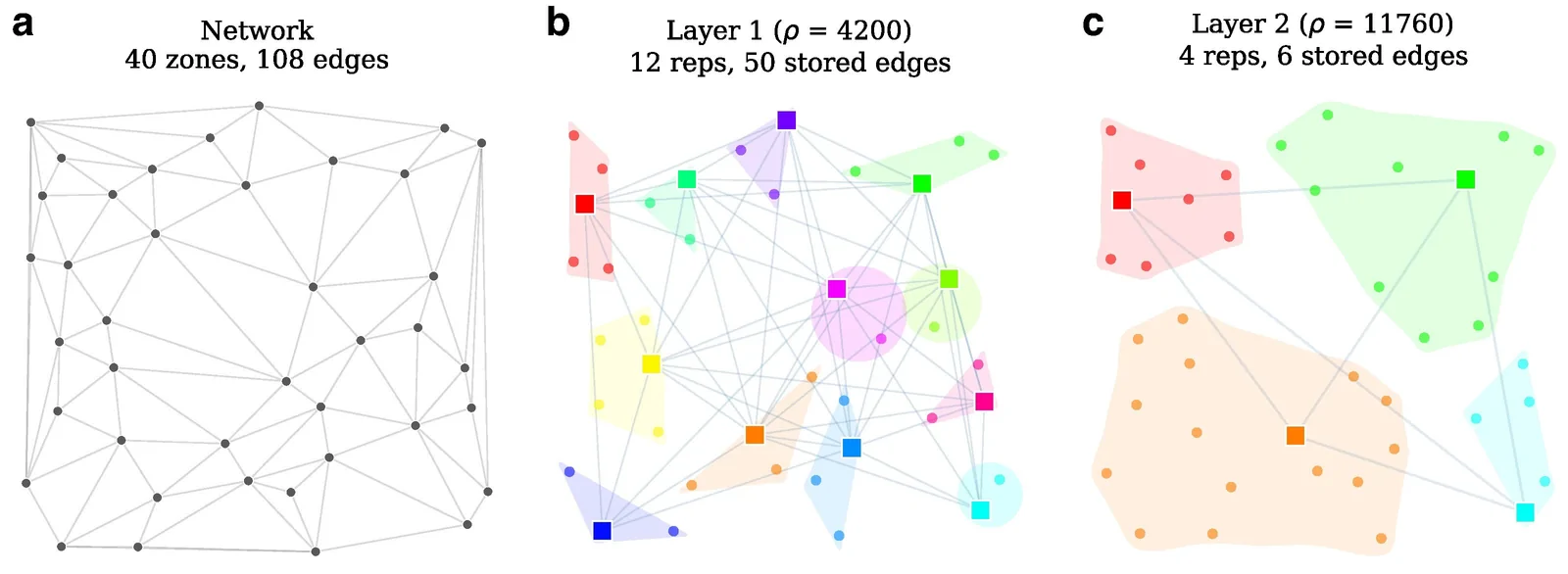
Hierarchy construction on a 40-zone random spatial network. a) Input graph. b) Layer 1 (ρ=4,200): 12 representatives (squares), each heading a group (shaded region). c) Layer 2 (ρ=11,760): 4 representatives with coarser groups. Stored edges connect representative pairs within the cutoff $\alpha \cdot \rho_{k}$.

### The hierarchical operator

Applying the operator to a vector x proceeds in three steps at each layer *k*:

1. Aggregate: $x_{k}=G_{k}\;x$
2. Interact: $c_{k}=(F_{k}-{\mathrm{Corr}}_{k})\;x_{k}$
3. Expand: $h=\sum_{k}G_{k}^{⊤}c_{k}$

This corresponds to the operator


(4) $$H=\sum_{k}G_{k}^{⊤}(F_{k}-{\mathrm{Corr}}_{k})\;G_{k},$$

which requires O(nlogn) operations under the assumptions in Complexity analysis section. The correction matrices ${\mathrm{Corr}}_{k}$ are needed because a node pair (i,j) may be represented at multiple layers: if nodes *i* and *j* belong to representatives that are close enough to appear in both a fine layer and a coarser layer, both $F_{k}$ and $F_{k^{\prime }}$ will contain a nonzero entry for the corresponding representative pair. Simply summing $G_{k}^{⊤}F_{k}G_{k}$ over all layers would double-count such pairs; ${\mathrm{Corr}}_{k}$ removes this redundancy. Each ${\mathrm{Corr}}_{k}$ is constructed as follows: for each nonzero entry (r,s) in $F_{k}$, we look up their representatives at each coarser layer $k^{\prime }$. If the coarser pair $\left(r^{\prime },s^{\prime }\right)$ also has a stored interaction in $F_{k^{\prime }}$, we set ${\mathrm{Corr}}_{k}[r,s]=F_{k^{\prime }}[r^{\prime },s^{\prime }]$, taking the value from the nearest coarser layer. Because we iterate only over the nonzero entries of the sparse matrices $F_{k}$, the total work is $O(E_{\mathrm{total}}\cdot K)$, where $E_{\mathrm{total}}$ is the total number of stored interactions across all layers.

The effect is that each layer contributes only the *difference* between its own interaction value and the next coarser layer’s value for the same pair. These differences telescope across layers, so that only the finest-layer value survives.

Proposition 1 **(Exactly once contribution)**

For every node pair (i,j) that appears in at least one layer, let $\hat{k}$ be the finest layer at which the representative pair $\left(\hat{i},\hat{\;j})=(r_{\hat{k}}[i],r_{\hat{k}}[j]\right)$ has a stored interaction in $F_{\hat{k}}$. Then (i,j) contributes exactly $f(c_{\hat{i}\hat{\;j}})$ to *H*—once, at the finest available resolution.

Proof. Let $\hat{k}<k_{1}<\cdots <k_{m}$ be the layers at which the representative pair for (i,j) has a stored interaction, ordered from finest to coarsest. At layers not in this set, the pair is absent from $F_{\ell }$ and contributes zero. At each layer in the set, the correction subtracts the nearest coarser layer’s value:

- at the coarsest layer $k_{m}$, no coarser layer stores the pair, so ${\mathrm{Corr}}_{k_{m}}=0$ and the contribution is $F_{k_{m}}$;
- at each finer layer $k_{j}$ (j<m), the correction subtracts the value from $k_{\;j+1}$, giving contribution $F_{k_{j}}-F_{k_{\;j+1}}$.

Summing across all layers in the set:

$$(F_{\hat{k}}-F_{k_{1}})+(F_{k_{1}}-F_{k_{2}})+\cdots +(F_{k_{m-1}}-F_{k_{m}})+F_{k_{m}}=F_{\hat{k}},$$

where each $F_{\ell }$ is shorthand for $F_{\ell }[r_{\ell }[i],r_{\ell }[j]]$. The telescoping sum yields exactly the finest-layer value, contributed once.

While one could look up $\hat{k}$ for each pair individually, the correction formulation (4) decomposes the operator into sparse matrix products that can be evaluated layer by layer, enabling efficient matvec without per-pair lookup.

## Complexity analysis

In this section, we summarize the computational work required to build and apply the hierarchical operator, and to update it after local changes to the underlying network. We emphasize that these estimates are conditional on structural assumptions that hold in many spatial and transport networks, but not in all graphs.

We denote:

- *n* = number of nodes (zones),
- *m* = number of edges,
- *K* = number of hierarchical layers,
-
$W_{k}$ = average work for a cutoff-limited shortest-path search at layer *k* (cutoff $\alpha \cdot \rho_{k}$),
-
$d_{\mathrm{avg}}$ = average number of stored neighbors per representative (sparsity factor),
- *Δ* = number of origin–destination pairs for which a local change improves the shortest path (“impact size”).

### Structural assumptions

We do not claim worst-case guarantees over all graphs. Our analysis and design are tailored to sparse, roughly spatial networks (28) of the kind found in transport and spatial interaction modeling. In particular, we assume:

1. Sparse network: the graph is sparse, with m=O(n), and running Dijkstra’s algorithm from a single source requires O(mlogn)≈O(nlogn), or better with specialized implementations.
2. Controlled local growth: the number of nodes within a ball of radius *r* around any node grows at most polynomially in *r* (for example, as in road networks embedded in low-dimensional space). This alone does not bound coarse-layer search volume; the near-linear overall scaling arises because the number of sources $S_{k}$ shrinks at the same rate that per-source work $W_{k}$ grows, keeping the product $S_{k}\cdot W_{k}$ roughly constant across layers (see below).
3. Bounded representative degree: at each layer *k*, the number of stored interactions per representative, $d_{\mathrm{avg}}$, remains bounded or grows slowly with *n*, due to the use of cutoffs and the decay of *f* with distance.
4. Logarithmic depth: the number of layers *K* grows slowly with system size, typically K=O(logn) when radii $\rho_{k}$ increase geometrically.

Under these assumptions we expect near-linear or nlogn scaling. The assumptions can fail—for dense graphs, or for networks with abundant long-range shortcuts—and then the work can be higher. Importantly, such structures are not mere mathematical artifacts: hub-and-spoke systems common in transport (public transport, airline, or micromobility networks) concentrate long-range connectivity in a few nodes and can violate the controlled-growth assumption. We return to this limitation in Discussion section.

### Building the hierarchy

Constructing the hierarchy involves selecting representatives $R_{k}$ at each layer *k* and computing shortest-path costs from each representative to other nodes within $\alpha \cdot \rho_{k}$. If $S_{k}$ is the number of sources at layer *k*, each requiring work $W_{k}$, the total work is


$$T_{\mathrm{hierarchy}}=\sum_{k}S_{k}\cdot W_{k}.$$

Because $S_{k}$ decreases as *k* increases while $W_{k}$ grows, $S_{k}\cdot W_{k}$ remains roughly constant across layers, yielding $T_{\mathrm{hierarchy}}\approx O(n\cdot K_{\mathrm{eff}})$, where $K_{\mathrm{eff}}$ captures the effective number of representative runs. Empirically, behavior is close to linear in *n*.

### Building interactions and corrections

Transforming costs $C_{k}$ into interactions $F_{k}$ and constructing correction matrices ${\mathrm{Corr}}_{k}$ requires iterating over stored representative pairs. The total number of stored entries is $E_{\mathrm{total}}=\sum_{k}E_{k}=O(n\cdot K\cdot d_{\mathrm{avg}})$, since $E_{k}\propto |R_{k}|\cdot d_{\mathrm{avg}}$ and $\left|R_{k}\right|$ shrinks with *k*.

Correction construction visits each fine-layer entry and searches coarser layers until a correction is found. In the worst case each entry is visited *K* times, giving $T_{\mathrm{interaction}}=O(n\cdot K^{2}\cdot d_{\mathrm{avg}})$; in practice early termination brings this closer to $O(n\cdot K\cdot d_{\mathrm{avg}})$. With K≈logn and modest $d_{\mathrm{avg}}$, the effective scaling is O(nlogn).

### Applying the operator

Applying the operator *H* to a vector x consists of:

1. aggregating x to representatives at each layer: $x_{k}=G_{k}\;x$,
2. computing $c_{k}=(F_{k}-{\mathrm{Corr}}_{k})\;x_{k}$, and
3. expanding and summing: $h=\sum_{k}G_{k}^{⊤}c_{k}$.

Aggregation and expansion each require O(n⋅K) work; the dominant cost is the sparse matrix–vector multiplies whose total nonzeros are $E_{\mathrm{total}}$. Thus:


(5) $$T_{\mathrm{apply}}=O(E_{\mathrm{total}})+O(n\cdot K)\approx O(n\cdot K\cdot d_{\mathrm{avg}}).$$

With K≈logn and modest $d_{\mathrm{avg}}$, $T_{\mathrm{apply}}\approx O(n\log\;n)$ in the transport-like networks that motivate our work.

### Dynamic updates

The hierarchy admits an incremental update framework in which update propagation is proportional to the number of affected pairs times the hierarchy depth, rather than to overall network size. A detailed analysis is provided in the Supplementary material.

### Summary

Under standard sparsity and growth assumptions for spatial and transport networks, building and applying the operator scale as O(nlogn). Once affected pairs are identified, update propagation scales with Δ⋅K rather than with *n* (Supplementary material). We make no universal worst-case guarantees; our claims are intended for sparse, geographically embedded networks.

## Approximation error

The hierarchical operator is an approximation to the dense interaction kernel defined by (1), where $c_{ij}=c(i,j)$ is the exact shortest-path cost between nodes *i* and *j*. In this section, we outline the main sources of approximation error and how they relate to design parameters of the hierarchy.

At a high level, errors arise from two choices:

1. Grouping error: nodes within the same group at a given layer *k* share a representative $r_{k}[i]$. When we approximate interactions at that layer using representative costs $c(r_{k}[i],r_{k}[j])$ rather than the exact node-to-node costs c(i,j), we incur a discrepancy that depends on how far nodes deviate from their representatives.
2. Truncation across layers: interactions at a given scale are only stored within a cutoff range $\alpha \cdot \rho_{k}$. Beyond this range, interactions are delegated to coarser layers. This can slightly distort how interaction mass is partitioned across scales, but by construction each origin–destination pair is still accounted for at some layer, so we do not “drop” interactions entirely.

A simple way to quantify grouping error is to bound the diameter of groups. Suppose that at layer *k* the maximum distance between any node *i* and its representative $r_{k}[i]$ is at most $\delta_{k}$:


$$c(i,r_{k}[i])\leq \delta_{k}\;\forall \;i.$$

If the decay function *f* is Lipschitz-continuous^a^ with constant $L_{f}$, i.e. $\left|f(x)-f(y)|\leq L_{f}\cdot |x-y\right|$, then for any node pair (i,j) represented at layer *k* we have


(6) $$|f(c(i,j))-f(c(r_{k}[i],r_{k}[j]))|\leq L_{f}\cdot |c(i,j)-c(r_{k}[i],r_{k}[j])|.$$

A concrete bound on the distance discrepancy follows from the triangle inequality on the shortest-path metric. Since *c* satisfies the triangle inequality on undirected graphs with non-negative edge costs:


(7) $$|c(i,j)-c(r_{k}[i],r_{k}[j])|\leq c(i,r_{k}[i])+c(j,r_{k}[j])\leq 2\delta_{k}.$$

Combining (6) and (7) gives a per-entry error bound:


(8) $$|f(c(i,j))-f(c(r_{k}[i],r_{k}[j]))|\leq 2L_{f}\delta_{k}.$$

This bound is simple but always valid: the per-entry interaction error at layer *k* is controlled by twice the product of the Lipschitz constant of *f* and the maximum group radius $\delta_{k}$.

 

 

##### Near-field and far-field error regimes

For power-law decay $f(c)=(c+\delta_{0}{)}^{-\beta }$ the global Lipschitz constant $L_{f}=\beta \delta_{0}^{-\beta -1}$ can be very large, making (8) appear loose. The hierarchical structure resolves this naturally. At the finest layer (k=0) every node represents itself, so pairs resolved there incur *zero* grouping error. Pairs resolved at layer k≥1 were not within the cutoff at layer k−1, so their true costs exceed $\alpha \cdot \rho_{k-1}-2\delta_{k-1}$. In this range the relevant Lipschitz constant is not the global one but the maximum slope of *f* over large costs, which decreases rapidly as the decay function flattens. The bound (8) thus splits into a near-field regime (exact) and a far-field regime (bounded by the *local* smoothness of *f*). For exponential decay the global Lipschitz constant 1/λ is already bounded, and the distinction is less critical.

### Practical guidelines and empirical characterization

The bounds above yield two practical guidelines:

- Strongly distance-sensitive kernels (for example with very steep decay) have smaller effective $L_{f}$ in the relevant cost range, requiring less aggressive hierarchy parameters to keep errors low.
- Increasing the overlap factor *α* and choosing base radius $\rho_{0}$ commensurate with the network’s mean edge cost reduces group diameters at any given scale, at the cost of more computation and memory.

The bounds above control the *magnitude* of the error but not its *sign*. The sign structure matters in practice: because decay functions are convex, symmetric distance errors translate into interaction errors with a positive mean (Jensen’s inequality), so the operator systematically overestimates interactions in the far field. A second-order analysis of this bias is given in the Supplementary material, and Error anatomy of the national-scale case study section quantifies it empirically at national scale.

Because the theoretical bounds involve quantities ($\delta_{k}$, $L_{f}$) that depend on graph geometry, they are most useful as qualitative guides. We therefore complement them with systematic empirical error characterization: in Computational experiments section, we compare Hx against the dense baseline Fx across 1,800 parameter configurations on synthetic networks (error below 2.5% with $\rho_{0}=3\bar{c}$ and α=2, Table S3), and dissect distance-resolved error and bias on the full-scale GB case study (Error anatomy of the national-scale case study section).

## Computational experiments

We validate the operator’s scaling behavior and approximation quality through systematic benchmarks. All experiments were run on a single machine with an AMD Ryzen Threadripper PRO 7985WX processor (64 cores, 128 hardware threads) and 754 GB RAM, using Python 3.12, NumPy 2.4.2, SciPy 1.17.1, NetworkX 3.6.1, and the HierX package (v0.1.1). The hierarchical operator uses SciPy’s Dijkstra on compressed sparse row matrices with 32 parallel workers; the matrix–vector product is single-threaded. Synthetic networks have Euclidean edge costs in meters.

 

### Scaling performance

We measure build and matvec time for random spatial networks from n=100 to n=100,000 zones on a square of side $\sqrt{n}\times 5,000$ m. Here, $\bar{c}$ denotes the mean edge cost of the network, a characteristic local spacing scale; for these networks $\bar{c}\approx 10,000$ m. Parameters: $\rho_{0}=10,000\text{\textbackslash{}, m}\approx 1\bar{c}$, γ=2, α=1.5, $f(c)=(c+1,000{)}^{-2}$. Fitting t=a⋅nlogn yields $R^{2}=0.998$ for total build and $R^{2}=0.995$ for matvec, confirming O(nlogn) scaling (Table S1, Fig. 2). Activity vectors are uniform (x=1). At n=10,000 the hierarchical method is 30× faster than the dense $O(n^{2})$ baseline.

**Figure 2:**
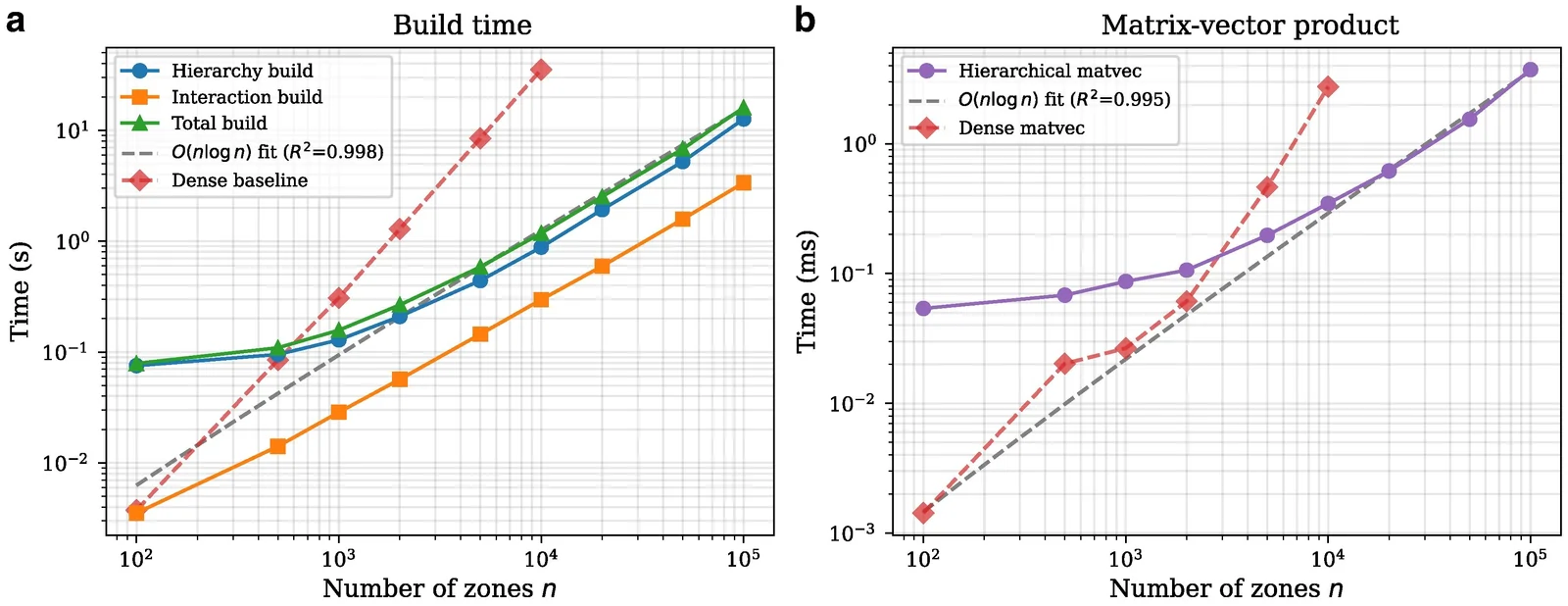
a) Build time vs. number of zones with O(nlogn) fit. b) Matvec time with dense baseline comparison.

### Approximation error vs. parameters

A systematic parameter sweep across 1,800 configurations (5 base radii × 5 overlap factors × 3 increase factors × 4 interaction functions × 6 networks) confirms that the overlap factor *α* is the primary accuracy knob: increasing *α* from 1.0 to 3.0 reduces median error by ∼7× (Table S2, Fig. 3). The joint dependence on base radius and overlap factor is shown in Fig. S1.

**Figure 3:**
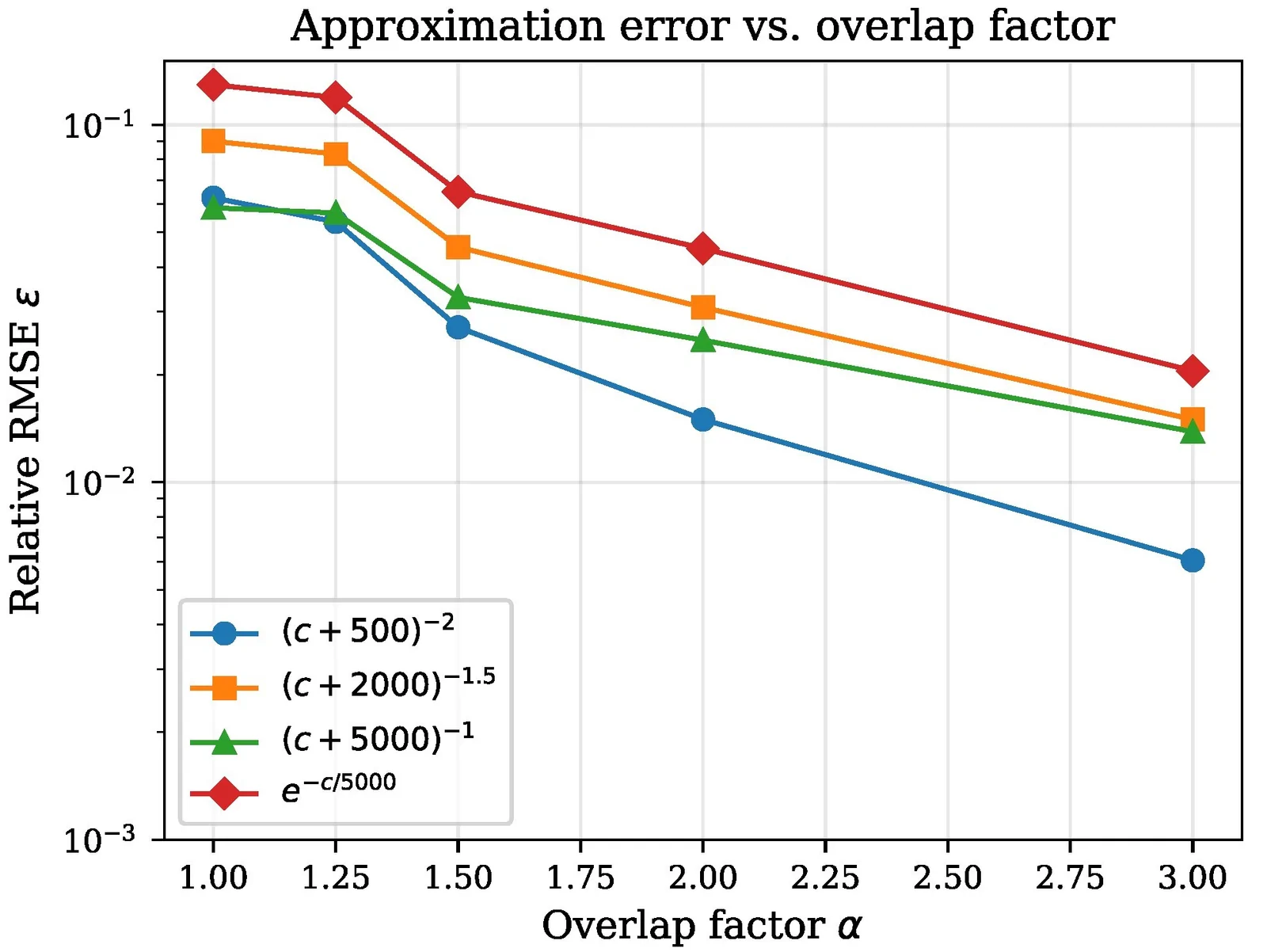
Relative RMSE ε vs. overlap factor for four interaction functions (20×20 grid, γ=2, $\rho_{0}=4,000$ m).

With $\rho_{0}=3\bar{c}$ ($\bar{c}\approx 10,000$ m, giving $\rho_{0}\approx 30,000$ m) and α=2.0, relative error remains below 2.5% up to n=5,000 (Fig. S2, Table S3). For the tested kernels, steeper decay generally gives lower error, consistent with the near-field/far-field analysis of Approximation error section (Fig. S3). The hierarchical representation achieves up to 10× compression in stored entries compared to the dense matrix (Fig. S4).

### Comparison with alternative methods

We compare the hierarchical operator with distance cutoff truncation and Nyström low-rank approximation on a 25,000-zone grid network (158×158, spacing 1,000 m, diameter 314 km). To compare methods independently of hardware and parallelization strategy, we report *total nodes explored*: the total number of finite-distance entries discovered across all Dijkstra calls during the build phase. For accuracy, we report the relative root-mean-square error (RMSE) $\varepsilon =\|h^{\mathrm{approx}}-h^{\mathrm{dense}}\|/\|h^{\mathrm{dense}}\|$. Activity vectors are drawn uniformly at random ($x_{j}∼U[0.1,1.1]$) to avoid bias from homogeneous loads.

Distance cutoff computes exact shortest paths within a radius (10, 25, or 50% of diameter) and zeros remaining entries. Nyström samples *m* landmarks, computes an n×m cross-similarity matrix *C* and approximates the full matrix as $CW^{+}C^{⊤}$ (9), where $W^{+}$ is the pseudoinverse of the m×m landmark submatrix. This is the standard (unmodified) Nyström formulation; indefinite-kernel extensions exist but were not tested. We use m=50 to 2,000.

Figure 4 shows the error-vs.-work Pareto frontier across four kernels. The hierarchical operator (α=2) explores ≈7.0 M nodes. On the steep kernel $(c+500{)}^{-2}$, it achieves 5.1% RMSE; Nyström fails (∼74% even at m=2,000) because the narrow support defeats sparse landmark sampling, while a 50% cutoff achieves 0.7% but requires 74× more work. On the moderate kernel $(c+2,000{)}^{-1.5}$, the hierarchical operator gives 6.9% RMSE; Nyström at m=2,000 reaches comparable error (6.5%) at 7× the cost. On the shallow kernel $(c+5,000{)}^{-1}$, Nyström is competitive—5.8% RMSE at m=200 (5.0 M nodes) vs. the operator’s 5.3%—and achieves 0.4% at higher landmark counts, the one regime where its low-rank assumption holds. On the exponential kernel $e^{-c/5,000}$, the operator gives 9.1% RMSE; cutoff at 10% diameter achieves 1.1% at 6× more work, while Nyström again fails (>57% error up to m=200).

**Figure 4:**
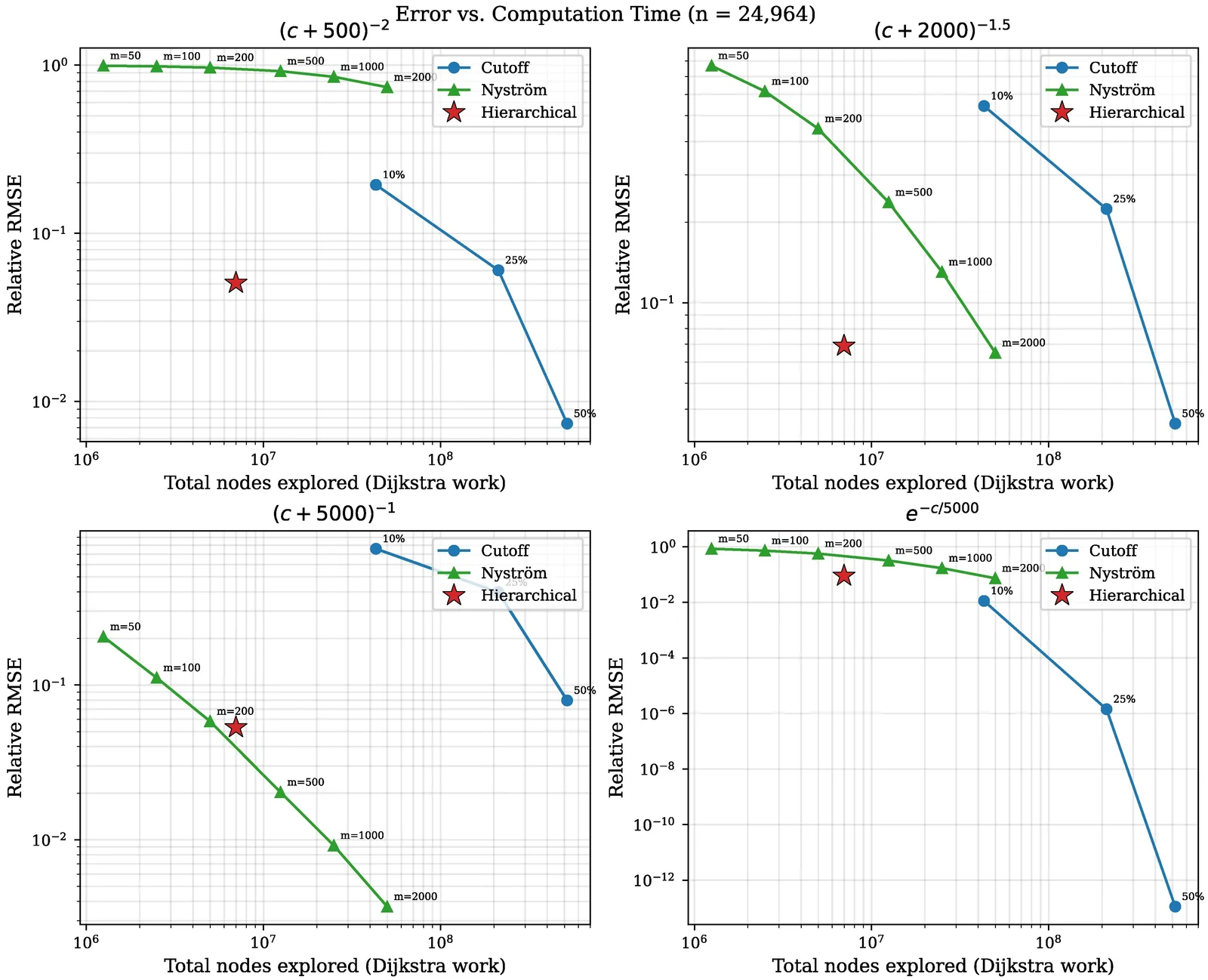
Error-vs.-computational-work Pareto frontier on a 25,000-zone grid network (spacing 1,000 m, diameter 314 km). The *x*-axis is total nodes explored (see text for definition); the *y*-axis is relative RMSE. Each panel corresponds to one interaction function. Cutoff variants are labeled by radius fraction of the network diameter; Nyström variants by landmark count *m*. The hierarchical operator (star) achieves competitive accuracy on steep kernels; on the smoothest kernel, Nyström dominates the error-work frontier.

### Application to real-world networks

To validate beyond synthetic benchmarks, we apply the operator to real road networks extracted from OpenStreetMap (OSM) (29). Two London networks are used: a 13 km-radius drive network centered on Charing Cross (n=61,432 nodes, $\bar{c}=9.1$ s, diameter 3,181 s) and a 5 km-radius walk network (n=70,095 nodes, $\bar{c}=25.8$ s). Edge costs are free-flow travel times (seconds) imputed from OSM speed limit tags.

On the driving network with steep decay $(c+300{)}^{-2}$, the hierarchical operator (α=2, $\rho_{0}=3\bar{c}$) achieves 8.0% RMSE while exploring only 15.8 M nodes—∼54× less Dijkstra work than the 25% cutoff, with 5× better accuracy. On the very steep kernel $(c+60{)}^{-2}$, Nyström fails: even m=1,000 landmarks yield 13.5% RMSE, while the hierarchical operator achieves 8.6% RMSE with ∼4× less work. On broader kernels, Nyström dominates the error-work tradeoff (Fig. S5); the hierarchical operator’s advantage is confined to steep decay functions where low-rank approximation breaks down.

The Nyström failure is even more pronounced on the walking network with $(c+60{)}^{-2}$, where the 60 s offset (≈83 m at walking speed) produces steep local gradients that sparse landmark sampling cannot capture. Even m=1,000 landmarks yield 50.2% RMSE. Distance cutoff at 50% diameter achieves 1.8% RMSE but explores 3.9 B nodes. The hierarchical operator at α=5 achieves comparable accuracy (2.5% RMSE) while exploring only 91.9 M nodes—43× less Dijkstra work. Figure 5 summarizes the London results.

**Figure 5:**
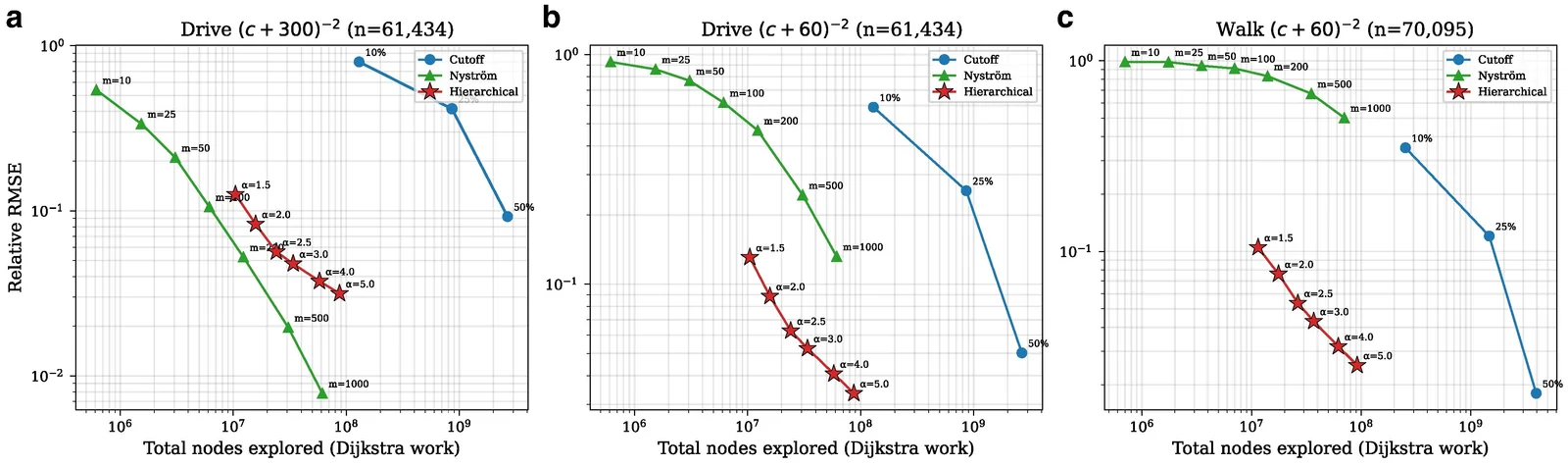
Error-vs-computational-work tradeoff on London networks. a) Drive network (n=61,432), steep decay $(c+300{)}^{-2}$. b) Drive network, very steep decay $(c+60{)}^{-2}$. c) Walking network (n=70,095), steep decay $(c+60{)}^{-2}$. The hierarchical operator (stars) achieves competitive accuracy at substantially lower computational cost than distance cutoffs (circles). Nyström (triangles) fails on the steep kernels. Full results for all four drive-network kernels are in Fig. S5.

#### Accessibility at metropolitan and national scales

To demonstrate the operator at scale, we compute population-weighted accessibility on the Great Britain driving network (2,576,491 nodes, 3,017,232 edges) and population- and employment-weighted accessibility on the London pedestrian network (1,765,539 nodes, 1,917,400 edges). Activity vectors are constructed by disaggregating Census Output Area population counts (30, 31) to individual building footprints in proportion to estimated floor area. For the London walking network, each building is snapped to its nearest network edge and its count split equally between the two endpoint nodes; for the GB driving network, each building is snapped to its nearest network node. Of the 65.0 million residents in the combined GB census, 59.7 million (91.7%) are successfully assigned to network nodes; the remainder reside in areas without matched OSM building footprints.

For the GB network, we use $f(c)=(c+300{)}^{-2}$ with offset matching a 5-min drive. The hierarchy (10 layers, $\rho_{0}=60$ s, α=2) builds in ∼4,300 s and stores 720 million interaction entries; each matvec completes in ∼700 ms. For the London walking network (7.7 million residents, 4.2 million workplace population), a steeper decay $f(c)=(c+60{)}^{-2}$ captures the shorter range of pedestrian interaction. The hierarchy (11 layers, $\rho_{0}=3\bar{c}$, α=2) builds in ∼2,000 s and stores 82 million interaction entries; each matvec completes in ∼150 ms. Computing dense ground truth for the full fields is impractical at these scales; accuracy is validated on the 61,432- and 70,095-node subgraphs (Application to real-world networks section) and, for the GB network, directly at full scale via sampled exact ground truth (Error anatomy of the national-scale case study section). In both cases, a single hierarchy build supports arbitrary activity vectors at marginal cost per evaluation.

Figure 6 shows the resulting maps. Figure 6a reveals the expected national pattern: major urban agglomerations appear as bright clusters, with accessibility tapering into rural and highland areas. Figure 6b and c compare population and employment accessibility on the London walking network: population accessibility reflects London’s distributed residential fabric, while employment accessibility peaks sharply in the City and West End.

**Figure 6:**
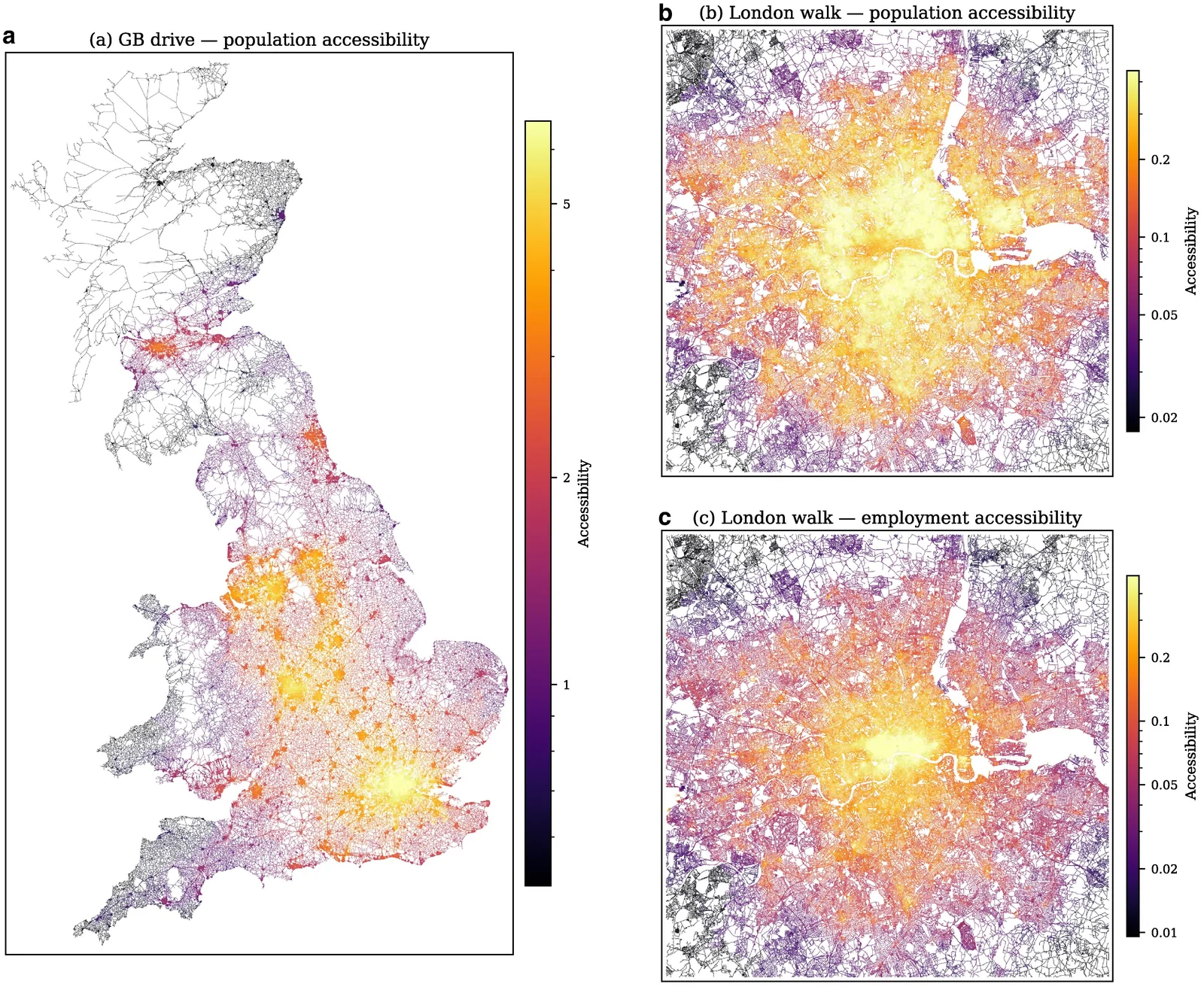
Accessibility maps computed by the hierarchical operator. a) Great Britain driving network (n=2,576,491): population-weighted accessibility with $f(c)=(c+300{)}^{-2}$. b, c) London pedestrian network (n=1,765,539): population and employment accessibility with $f(c)=(c+60{)}^{-2}$, walking speed 5 km/h. Edges are encoded by the mean accessibility of their endpoint nodes (log scale, per-panel normalization). Activity vectors are Census counts disaggregated to buildings by floor area. Each hierarchy is built once; subsequent matvec evaluations take ∼700 ms (GB) and ∼150 ms (London). An interactive zoomable version is available at https://hierx.github.io/hierx-paper/interactive_maps/index.html. Network data © OpenStreetMap contributors, available under the Open Database License. Census data: Office for National Statistics and National Records of Scotland, Crown copyright, Open Government Licence v3.0.

### Error anatomy of the national-scale case study

While dense ground truth for the full 2.58-million-node field is out of reach, *exact* values are computable at sampled nodes: each exact accessibility value $h_{i}$ requires only one full Dijkstra run from node *i*. We exploit this to dissect, at full national scale, how the operator’s error depends on distance, on the decay kernel, and on the activity distribution—and whether the error is systematically biased. We sample 1,000 origins uniformly at random, compute exact network distances to all nodes, and compare against the hierarchical effective cost (the finest-layer representative cost of Proposition 1) for 536,656 origin–destination pairs stratified across log-spaced distance bins. The hierarchy is rebuilt deterministically and reproduces the Fig. 6a accessibility field exactly, so these statistics characterize that map directly.

#### Distance error

Pairs within the finest-layer cutoff (≲2 min) are resolved exactly (Fig. 7a). Beyond it, the relative distance error is nearly scale-free: per-bin RMSE stays at ∼20% across three orders of magnitude of distance, with a small systematic underestimate (mean −2.6%). Because layer radii grow geometrically with distance, the *absolute* error grows proportionally to distance while the *relative* error remains roughly constant.

**Figure 7:**
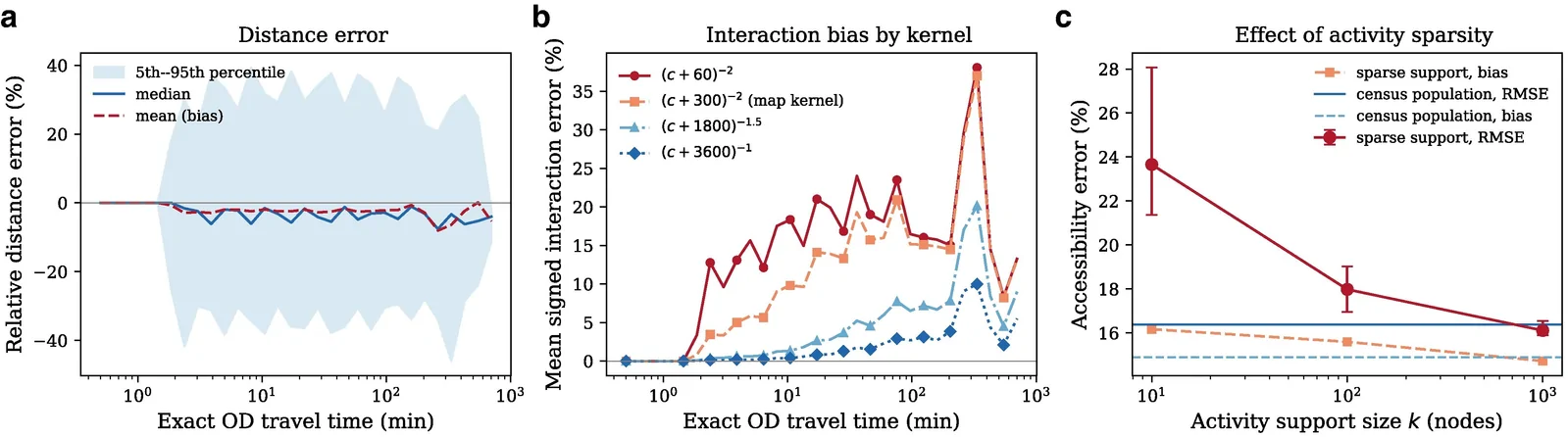
Error anatomy of the GB case study (n=2,576,491, α=2, $\rho_{0}=60$ s), against exact Dijkstra ground truth at 1,000 sampled origins. a) Relative distance error vs. exact OD travel time (536,656 sampled pairs): exact in the near field, then a nearly scale-free ∼20% spread with a small negative bias. b) Mean signed interaction error by distance bin for four kernels: convexity turns distance spread into systematically positive interaction bias, increasing with kernel steepness. c) Accessibility error vs. activity support size *k* (unit activity on *k* population-weighted nodes; exact full-network ground truth from *k* Dijkstra runs; whiskers span three trials): sparsity adds variance while the bias matches the census-population level (horizontal lines).

#### Interaction bias

These distance errors translate into interaction errors asymmetrically: decay kernels are convex, so by Jensen’s inequality even unbiased distance errors inflate the expected interaction. A second-order expansion for $f(c)=(c+\delta_{0}{)}^{-\beta }$ at $c\gg \delta_{0}$ predicts a mean relative interaction error of $\approx \frac{1}{2}\beta (\beta +1)\sigma^{2}-\beta \mu$, where *σ* and *μ* are the standard deviation and mean of the relative distance error in the far field (pairs beyond the finest-layer cutoff $\alpha \rho_{0}$, where grouping first occurs; Supplementary material). With σ≈0.20 and μ≈−0.027, this predicts biases from +6.5% (β=1) to +17% (β=2), in good agreement with the observed far-field bias (Fig. 7b) and with the accessibility-level bias at the sampled nodes: +3.7% for $(c+3600{)}^{-1}$, +7.2% for $(c+1800{)}^{-1.5}$, +14.9% for the map kernel $(c+300{)}^{-2}$, and +16.5% for $(c+60{)}^{-2}$ (Table S4). Note the reversal relative to small networks: when the kernel’s interaction mass fits within the finest exact layer ($\alpha \rho_{0}$), steeper kernels are *more* accurate (Approximation error vs. parameters section); at national scale the offsets place most mass beyond that cutoff, where convexity dominates, and steeper kernels are *less* accurate. Because these per-pair errors enter the accessibility sum weighted by interaction mass, the large far-field values in Fig. 7b contribute little—for the map kernel, pairs beyond 230 min carry only ∼1% of the mass.

The total error of the GB population-accessibility field (Fig. 6a) at the sampled nodes is ε=16.4%, and it decomposes into a largely uniform multiplicative inflation (total interaction mass ratio 1.139) plus dispersion: calibrating away the global factor reduces the error to 8.7%, and the rank correlation between approximate and exact accessibility is ρ=0.98. The systematic component is thus predictable in direction and magnitude, and applications sensitive to absolute levels should calibrate it away or increase *α*; the spatial pattern of the map is robust. Two mitigations from the adaptive-zoning literature also transfer to the hierarchy: replacing each fixed representative by a randomly drawn group member, which removes the bias at the cost of additional variance, or correcting representative costs by the ratio of Euclidean distances from the true and the representative origin to the destination (32).

#### Activity sparsity

For activity concentrated on *k* support nodes, exact ground truth for the *entire* network requires only *k* Dijkstra runs. Sweeping *k* with population-weighted support sampling (Fig. 7c, Table S5), the full-network RMSE falls from 23.7% at k=10 to 16.1% at k=1,000, approaching the 16.4% of the ubiquitous census population vector (59.7 M persons spread over 1.77 M nodes), while the bias stays at ≈+15% throughout. Sparse activity layers—accessibility to a small set of destinations such as airports, ports, or major hospitals—thus add variance, not bias: error concentrates where few support nodes are nearby, as their far-field contributions pass through coarse layers, while the systematic component is a property of the kernel and the distance distribution alone. When the important locations are known in advance, this residual is avoidable: resolving them at the finest layer—storing their exact interactions directly, which the correction matrices reconcile with the coarser layers by construction (Proposition 1)—makes their contributions exact, at a cost of one shortest-path solve and one dense row per designated location.

## Discussion

The hierarchical operator approximates global interactions on large networks by exploiting the same multiscale structure that makes these systems tractable in practice: local detail where it matters, coarser resolution where it does not. Evaluation runs in quasilinear time; the hierarchy suggests an incremental update framework in which update propagation is proportional to the number of affected pairs *Δ* times the hierarchy depth *K* (Supplementary material).

The approach targets a specific problem class—shortest-path decay kernels on general graphs—common in spatial interaction modeling but not directly addressed by existing hierarchical matrix or fast multipole methods. In settings with dense connectivity, rapidly changing global shortcuts, or kernels that do not decay with distance, other techniques may be more appropriate.

### When is the hierarchical operator the right choice?

Simple distance cutoffs suffice when the decay function is steep enough that 99% of interaction mass lies within a modest radius, and for small networks ($n<10^{3}$) dense computation is fast and exact. Efficient shortest-path query structures (22–24) accelerate individual lookups but do not directly provide the aggregated vector h=Hx. Nyström approximation (9) and random Fourier features (8) assume positive semi-definite kernels, which shortest-path interaction kernels on general graphs generally are not. The hierarchical operator is most advantageous when: (i) the network is large ($n≳10^{4}$); (ii) the decay function is shallow enough that distant interactions carry meaningful mass; (iii) the application requires repeated evaluations of Hx for varying x, amortizing build cost; and (iv) the network may evolve, making precomputed static structures expensive to maintain. Our baseline comparison (Comparison with alternative methods section) confirms this: the hierarchical operator achieves 5–9% RMSE; matching this accuracy with distance cutoffs requires ∼30× more Dijkstra work, and exceeding it can cost up to 74× more. Nyström approximation is competitive only for the smoothest kernels; for steep decay it exhibits ∼74% RMSE even at m=2,000 landmarks, a limitation compounded by network interventions that move the distance structure further from any Euclidean embedding.

### Parameter sensitivity

The overlap factor *α* is the most important tuning parameter, with a ∼7× error reduction from α=1.0 to α=3.0 (Table S2). The base radius $\rho_{0}$ should be at least 3× the mean edge cost; with $\rho_{0}=3\bar{c}$ and α=2, error remains below 2.5% up to n=5,000 (Table S3). The increase factor γ=2 balances resolution and computational cost.

### Topology effects

Regular grids show the most predictable behavior; random spatial networks exhibit higher error variance. The London experiments (Application to real-world networks section) confirm that the operator handles organic, irregular topology well, achieving 8% RMSE at α=2 with ∼54× less Dijkstra work than a 25% cutoff. Practitioners should calibrate against dense baselines on representative subnetworks before full-scale deployment.

### Interaction function dependence and bias

The effect of kernel steepness is scale-dependent. On small and moderate networks, steeper decay functions produce *lower* error, because interactions concentrate in nearby zones captured exactly by the finest layer. At national scale the ordering reverses (Error anatomy of the national-scale case study section): the error budget is carried by far-field pairs, where the convexity of the decay function converts the roughly symmetric distance error into a systematically *positive* interaction bias that grows with steepness ($\approx \frac{1}{2}\beta (\beta +1)\sigma^{2}$ for power-law exponent *β* and relative distance spread *σ*). Practitioners should expect the operator to overestimate accessibility on average, by a predictable margin that can be calibrated away via the total interaction mass, and should increase *α* when absolute levels matter.

### Hub-dominated and directed networks

The structural assumptions of Complexity analysis section can be violated by networks that are entirely realistic in transport analysis, not only by mathematical artifacts. Hub-and-spoke systems—public transport with express services, airline networks, micromobility with docking stations—concentrate long-range connectivity in a few nodes, so that the ball of reachable nodes around a hub grows much faster than polynomially in radius. This inflates the per-source search work $W_{k}$ and the stored degree $d_{\mathrm{avg}}$, eroding the complexity advantage, and shortcut-rich metrics also blur the separation of scales on which the grouping rests. A partial mitigation is that the hierarchy operates on the network metric itself rather than on a Euclidean embedding, so it degrades gracefully where Euclidean-based methods break outright; hub-aware representative selection (placing representatives at hubs) is an open direction. Directed graphs (one-way streets, bus routes) pose a separate, structural limitation: the current formulation assumes symmetric costs, since grouping and correction use a single cost per pair. Supporting asymmetric costs would require separate forward and backward cost structures (see Supplementary material).

### Limitations

The open-source HierX package does not yet include dynamic update functionality; the complexity analysis (Complexity analysis section) establishes an O(Δ⋅K) update framework, and preliminary prototype results support feasibility, but a full empirical validation will be reported separately. The Python implementation stores intermediate objects that inflate memory; the intrinsic sparse structure contains ∼10× fewer entries than the dense matrix, a compression advantage a compiled implementation would fully realize. Additional discussion of directed graphs, parallelization, and asymmetric costs is provided in the Supplementary material.

### Future directions

The most pressing next step is implementing and validating the dynamic update scheme on real infrastructure scenarios, including the impact-driven propagation theory outlined in Complexity analysis section. Representative selection could be improved via graph coarsening or centrality-based criteria. On the build side, contraction-hierarchy or hub-label queries could replace Dijkstra for the coarse layers, where few costs between widely separated representative pairs are needed (one-to-one queries), while cutoff-limited Dijkstra remains the right tool for the fine layers’ dense one-to-many searches; this division of labor could substantially reduce build-phase constants. On the accuracy side, the nested group structure invites $H^{2}$-inspired refinements (Connections to existing hierarchical and graph-based methods section): translating cost information between nested layers instead of recomputing it, and softening the hard single-representative assignment into a weighted average (convex combination) of nearby representatives, which in variable-order $H^{2}$-matrices improves approximation orders under certain conditions and could reduce the far-field grouping error here. Applying the operator to networks beyond transport—social, biological, or communication networks—would test the generality of the hierarchical approximation.

## Conclusion

This work establishes a hierarchical sparse-plus-correction operator as an alternative to dense distance-decayed interaction matrices on large sparse graphs. The operator adapts hierarchical interaction ideas from fast multipole methods, hierarchical matrices, and multiscale CA to shortest-path-based interaction kernels on general graphs.

Systematic benchmarks on networks up to 100,000 zones confirm O(nlogn) scaling for both build ($R^{2}=0.998$) and operator application ($R^{2}=0.995$). Application to real networks—a 2.58-million-node Great Britain driving network and a 1.77-million-node London walking network—demonstrates practical national- and metropolitan-scale performance: a one-time hierarchy build of roughly an hour yields an operator that evaluates each accessibility field in well under 1 s. With $\rho_{0}=3\bar{c}$ and α=2, relative RMSE remains below 2.5% up to n=5,000 (Table S3). Head-to-head comparison with distance cutoff truncation and Nyström low-rank approximation on a 25,000-zone network shows that, for steep and moderate decay kernels, it achieves 5–9% RMSE at a fraction of the Dijkstra work required by either baseline; for the smoothest kernels Nyström can achieve lower error at proportionally higher cost, but the hierarchical operator remains the most efficient option at comparable accuracy levels.

The complexity analysis establishes an incremental update framework in which update propagation is proportional to the number of affected pairs *Δ* times the hierarchy depth *K*, rather than to overall network size. A full empirical validation of this framework is deferred to future work.

The open-source HierX package, archived datasets, and Docker-based reproducibility pipeline accompany the article.

## Materials and methods

This study is computational. The hierarchical operator and its construction are specified in the *Methods: the hierarchical operator* section, and its cost and accuracy are analyzed in the *Complexity analysis* and *Approximation error* sections. Benchmark networks, hardware and software versions, parameter settings, and error metrics are given in the *Computational experiments* section, which also describes the sampled exact ground truth used for the national-scale case study. Code, data, and scripts that reproduce all results are listed under *Data Availability.*

## Supplementary Material

pgag317_Supplementary_Data

## Data Availability

The HierX Python package implementing the hierarchical operator is available as open-source software at https://github.com/hierx/hierx. Benchmark scripts, precomputed results, and data pipelines needed to reproduce every figure and table are provided in the companion repository at https://github.com/hierx/hierx-paper. A self-contained Docker image allows full reproduction of all figures and benchmarks without local installation; see the repository README for instructions. The London and Great Britain network snapshots, precomputed accessibility arrays, and Census-derived node activity vectors used in Accessibility at metropolitan and national scales section are archived on Zenodo (https://doi.org/10.5281/zenodo.19062193) for long-term reproducibility. These files can also be regenerated from OpenStreetMap (29) using the included scripts, though OSM data may change over time. Census 2021 population (TS001) and workplace (WP001) data for England and Wales are sourced from the Office for National Statistics (31) under the Open Government Licence; Scotland Census 2022 population data are sourced from National Records of Scotland (30).

## Notes

a The Lipschitz assumption effectively requires *f* to have no singularity at zero cost; all standard decay functions with a positive offset satisfy it.
