# Supplementary material for: HierX: Fast multiscale distance-decay interaction on million-node networks
Source: PNAS Nexus. 2026 Sep 17;5(9):pgag317. doi: 10.1093/pnasnexus/pgag317 (PMC13623006; doi:10.1093/pnasnexus/pgag317)
Supplement: Supplementary file 1 [file pgag317_supplementary_data.pdf]

Supplementary Information: HierX: Fast Multi-Scale Distance-Decay Interaction on Million-Node Networks

Alexander Hellervik, Joakim Bohlin, Claes Andersson

This Supplementary Information provides extended analyses supporting the main text. SI Figs. S1–S4 present tuning sensitivity and compression diagnostics; SI Fig. S5 shows the full London error-vs-work Pareto frontier. SI Tables S1–S3 list raw benchmark values quoted in the main text; SI Tables S4–S5 report the error anatomy of the Great Britain case study (Section 6 of the main text). The remaining sections extend the operator-error analysis (including a second-order analysis of the systematic interaction bias), discuss dynamic updates, and provide additional notes on directed graphs and parallelization.

SI Figures

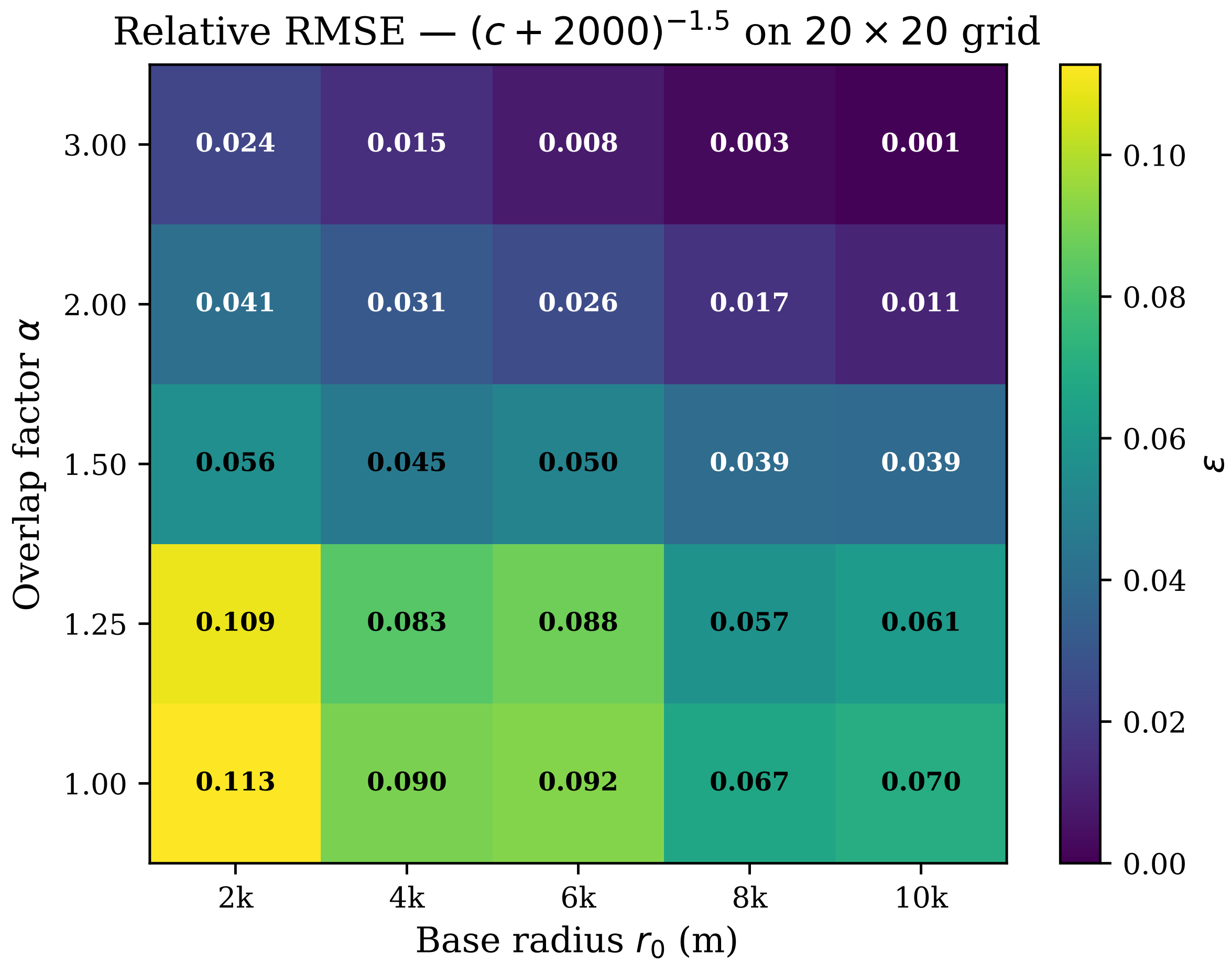

SI Figure S1. Relative RMSE ε as a function of base radius and overlap factor for the moderate interaction function $f(c) = (c + 2000)^{-1.5}$ on a 20×20 grid.

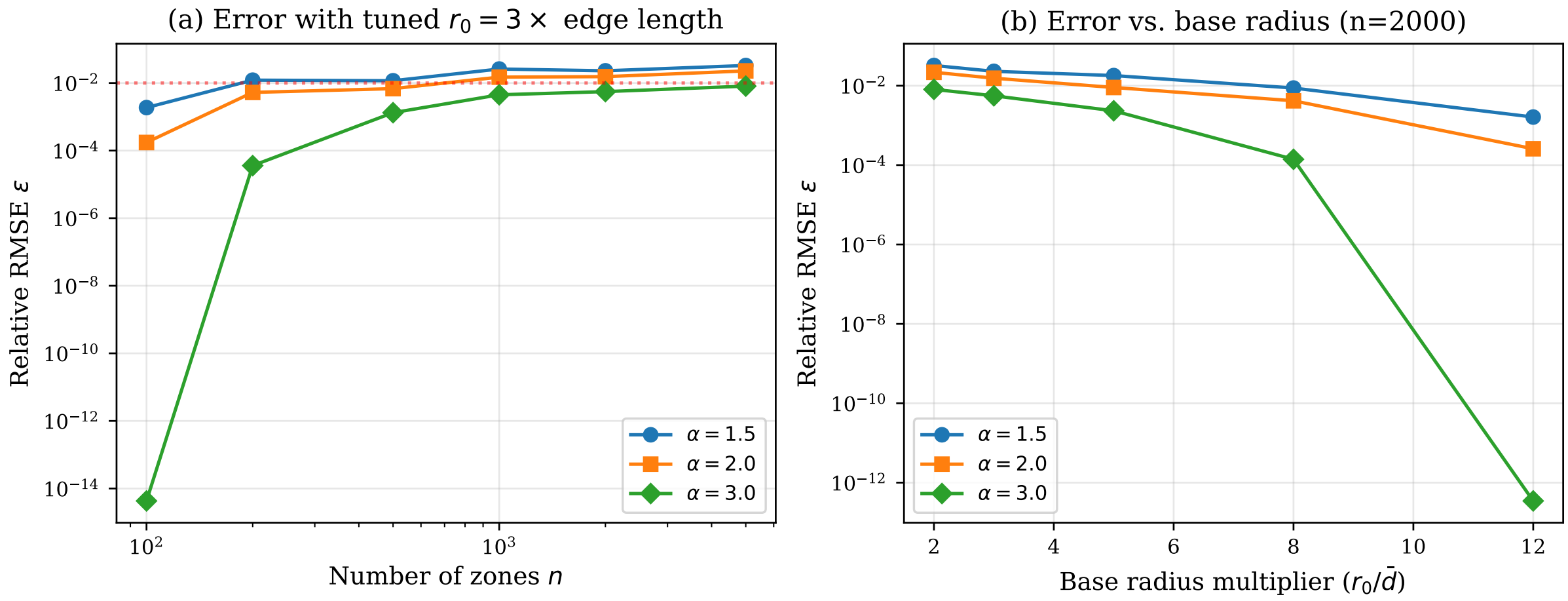

SI Figure S2. (a) Error vs. network size with tuned base radius. (b) Error vs. base-radius multiplier for different overlap factors.

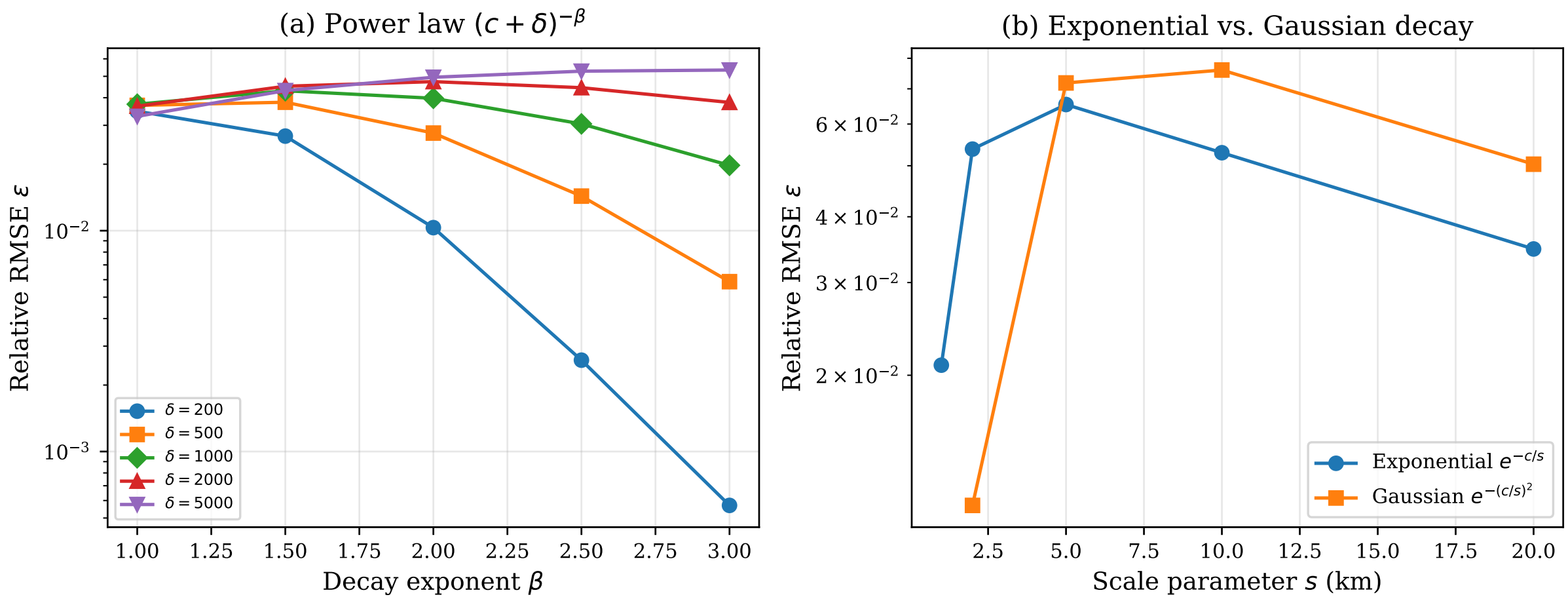

SI Figure S3. (a) Power law error vs. decay exponent β for different offsets δ ($\alpha = 1.5$, 20×20 grid). (b) Exponential vs. Gaussian error across length scales.

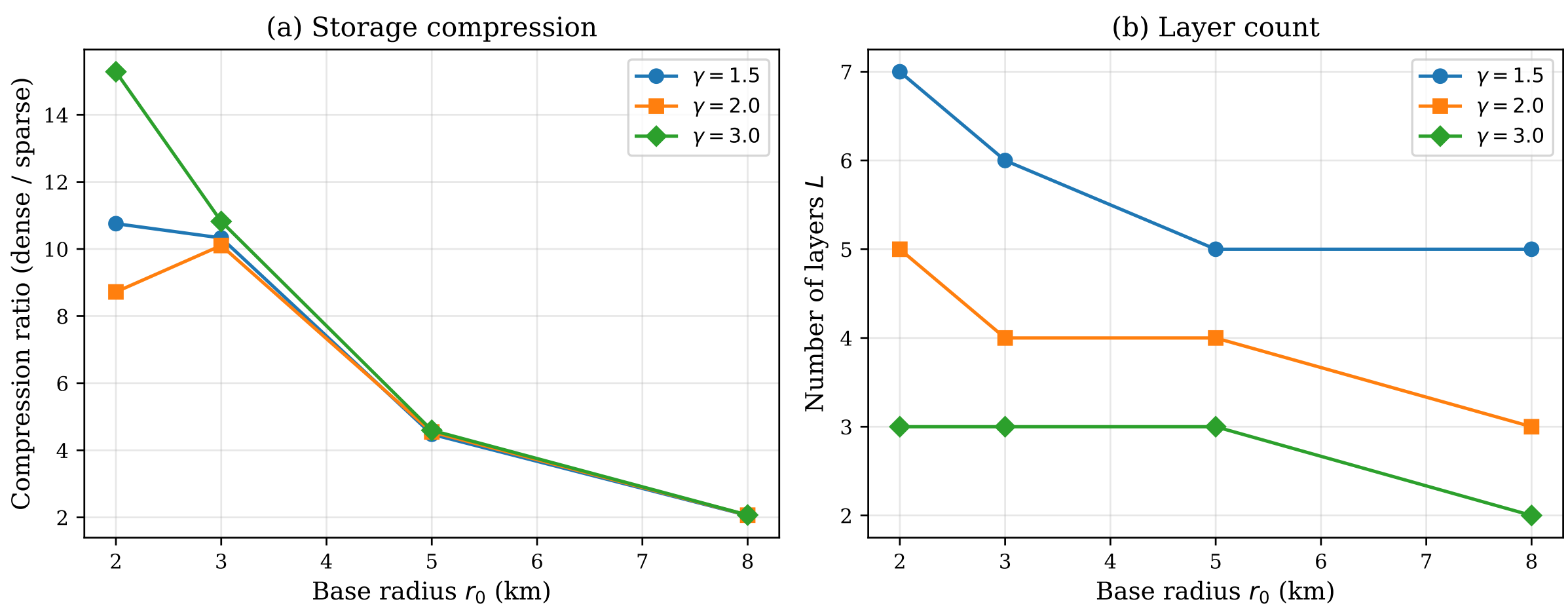

SI Figure S4. (a) Compression ratio vs. base radius for different increase factors. (b) Number of hierarchical layers vs. base radius.

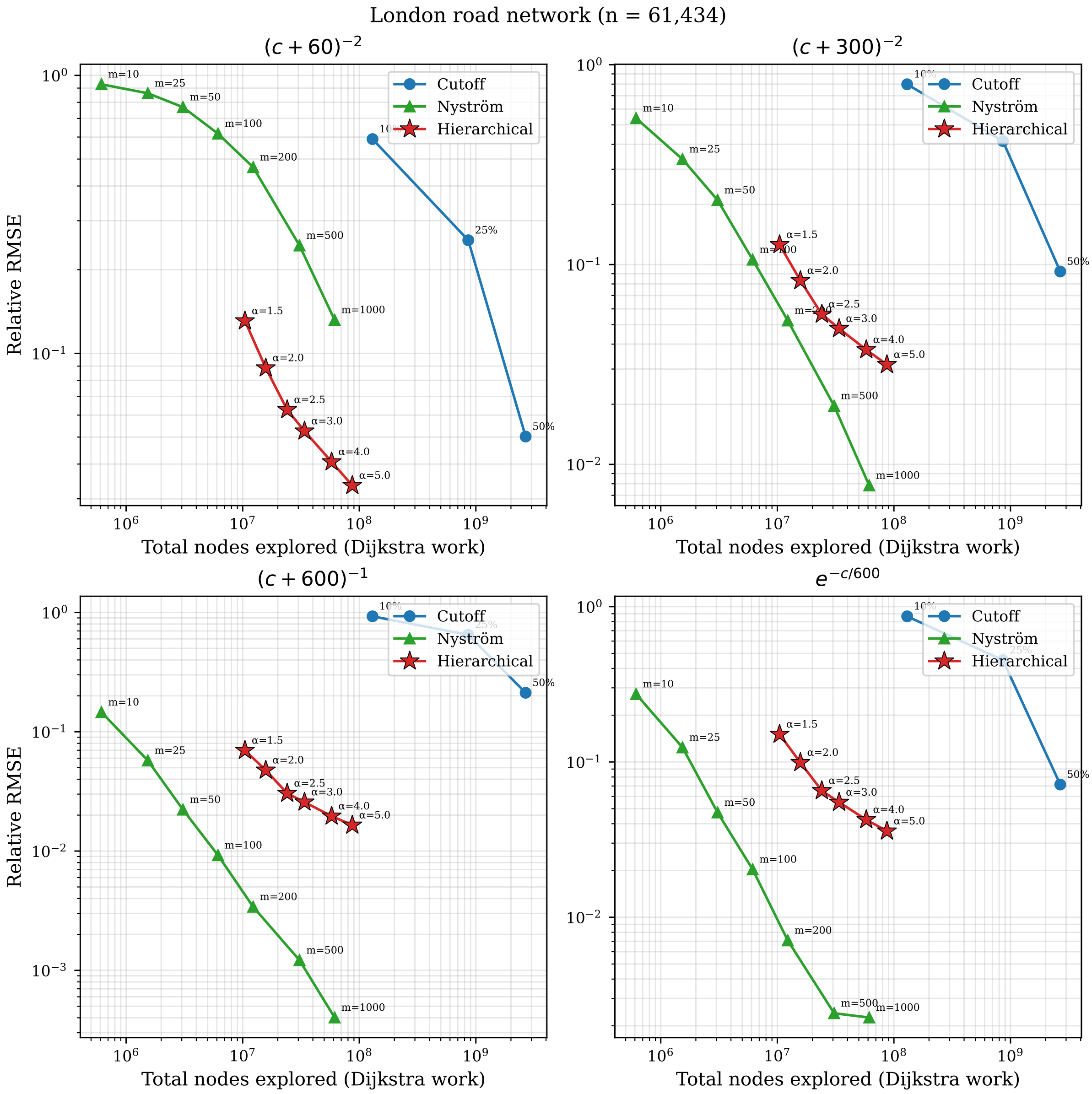

SI Figure S5. Error-vs-computational-work tradeoff on the London road network ($n = 61{,}432$) for four interaction functions. All methods use SciPy's Dijkstra backend. The x-axis is total nodes explored; the y-axis is relative RMSE. The hierarchical operator (stars, labelled by overlap factor α) explores 9.2–87.3 M nodes. On broader kernels, Nyström (triangles, labelled by landmark count m) dominates the error-work frontier; on the steep and very steep kernels, the hierarchical operator offers the best accuracy per unit of work. Nyström fails on $(c + 60)^{-2}$, where even $m = 1{,}000$ landmarks yield 13% RMSE. Distance cutoff (circles) explores 0.1–2.7 B nodes.

SI Tables

SI Table S1. Scaling performance of the hierarchical operator on random spatial networks (mean edge cost $\bar{c} \approx 10{,}000\,\text{m}$). Parameters: $\rho_0 = 10{,}000\,\text{m} \approx 1\bar{c}$, $\gamma = 2$, $\alpha = 1.5$, $f(c) = (c + 1000)^{-2}$. Dense baseline measured only for $n \leq 10{,}000$. Times are median of 5 trials; matvec is median of 5×10 repetitions. Hierarchy build uses 32 parallel Dijkstra workers (SciPy backend).

n	edges	K	t_{hier} (s)	t_{inter} (s)	t_{mv} (ms)	t_{dense} (s)	speedup
100	1,028	3	0.08	0.00	0.05	0.00	0×
500	6,206	4	0.10	0.01	0.07	0.08	1×
1,000	13,029	5	0.13	0.03	0.09	0.31	2×
2,000	26,683	5	0.21	0.06	0.11	1.29	5×
5,000	68,161	6	0.44	0.14	0.20	8.46	14×
10,000	139,028	7	0.88	0.30	0.35	35.26	30×
20,000	278,498	7	1.93	0.60	0.62	—	—
50,000	698,505	8	5.22	1.58	1.55	—	—
100,000	1,402,413	8	12.64	3.37	3.73	—	—

SI Table S2. Approximation error (relative RMSE ε) vs. overlap factor α. Statistics computed across all 360 parameter configurations (5 base radii × 3 increase factors × 4 interaction functions × 6 networks with edge costs in meters) for each overlap factor.

α	median ε	mean ε	10th pctile	90th pctile
1.00	0.059	0.066	0.010	0.129
1.25	0.056	0.064	0.007	0.123
1.50	0.048	0.051	0.004	0.102
2.00	0.029	0.032	0.002	0.065
3.00	0.009	0.014	0.000	0.035

SI Table S3. Scaling with properly tuned parameters. $\rho_0 = 3\bar{c}$ ($\bar{c} \approx 10{,}000\,\text{m}$), $\alpha = 2.0$, $\gamma = 2$. Error is relative RMSE ε.

n	ε	t_{hier} (s)	t_{mv} (ms)
100	< 0.001	0.065	0.04
200	0.005	0.082	0.05
500	0.007	0.136	0.11
1,000	0.015	0.276	0.20
2,000	0.015	0.549	0.43
5,000	0.023	0.965	1.22

SI Table S4. Accessibility error of the hierarchical operator at 1,000 uniformly sampled nodes of the GB driving network (n = 2,576,491, $\alpha = 2$, $\rho_0 = 60\,\mathrm{s}$), against exact Dijkstra ground truth. Bias, p5, and p95 are per-node signed relative errors; positive bias means the operator overestimates accessibility. ε is the norm-weighted relative RMSE $\|\mathbf{h}^{\mathrm{hier}} - \mathbf{h}^{\mathrm{exact}}\|/\|\mathbf{h}^{\mathrm{exact}}\|$ over the sample; $\varepsilon_{\mathrm{cal}}$ is the same after dividing $\mathbf{h}^{\mathrm{hier}}$ by the mass ratio $\sum h^{\mathrm{hier}}/\sum h^{\mathrm{exact}}$.

activity	kernel	bias	p5	p95	ε	mass	$\varepsilon_{\mathrm{cal}}$
population	$(c+300)^{-2}$	+14.9%	−3.2%	+37.1%	16.4%	1.139	8.7%
population	$(c+60)^{-2}$	+16.5%	−0.6%	+39.1%	17.7%	1.155	8.9%
population	$(c+1800)^{-1.5}$	+7.2%	−4.2%	+22.8%	9.3%	1.068	6.3%
population	$(c+3600)^{-1}$	+3.7%	−3.3%	+12.7%	5.5%	1.035	4.2%
workplace	$(c+300)^{-2}$	+14.2%	−3.9%	+37.0%	15.8%	1.131	9.0%
workplace	$(c+60)^{-2}$	+15.9%	−2.1%	+38.5%	16.8%	1.146	9.2%
workplace	$(c+1800)^{-1.5}$	+6.8%	−4.8%	+22.5%	9.1%	1.064	6.4%
workplace	$(c+3600)^{-1}$	+3.4%	−3.8%	+12.6%	5.4%	1.033	4.2%

SI Table S5. Effect of activity sparsity on accessibility error (GB driving network, kernel $(c+300)^{-2}$). For each support size k, unit activity is placed on k population-weighted sample nodes; exact ground truth for the *full* network is computed with k Dijkstra runs. RMSE is the norm-weighted relative error over all reachable nodes; mean over 3 trials (range in brackets). The census-population row is evaluated at the 1,000 sampled nodes of SI Table S4.

k	RMSE	bias	p95 |rel. err|
10	23.7% [21.4–28.1]	+16.2%	66.4%
100	18.0% [17.0–19.0]	+15.6%	46.6%
1000	16.1% [15.9–16.6]	+14.7%	37.6%
census pop.	16.4%	+14.9%	37.1%

Extended Error Analysis

Global operator error

The per-entry bound (Eq. 8 in the main text) can be lifted to the operator as a whole. Let $E = F - H$ denote the error matrix, where F is the dense interaction matrix and H the hierarchical approximation. By the exactly-once property (Proposition 1 in the main text), each node pair (i, j) is represented at exactly one layer $\hat{k}(i, j)$, so

$$|E_{ij}| = |f(c_{ij}) - f(c_{\hat{\imath}\hat{\jmath}})| \leq 2\,L_f\,\delta_{\hat{k}(i,j)}.$$

The worst-case entry error is therefore bounded by the coarsest group diameter:

$$\|E\|_{\max} \leq 2\,L_f\,\delta_K. \tag{1}$$

This bound is tight at the entry level but does not directly bound the matvec error $\|E\mathbf{x}\|_\infty$, which depends on group sizes and the distribution of pairs across layers. The hierarchical structure nonetheless keeps global error low through a separation of roles: nearby pairs, which carry the largest interaction values and are most sensitive to cost errors, are resolved at fine layers where δ_k is small; distant pairs are resolved at coarse layers where δ_k is large but the decay function is flat, suppressing the effective per-entry error (as discussed in the near-field/far-field analysis in the main text). We quantify the resulting accuracy empirically in the main text (Section 6).

Second-order analysis of the systematic interaction bias

The per-entry bound above is symmetric, but the realized interaction error is not: decay functions are convex, so distance errors inflate interactions on average. Let $\hat{c} = c\,(1+e)$ denote the hierarchical effective cost for a pair with exact cost c, where e is the relative distance error with mean μ and standard deviation σ across pairs at a given scale. For power-law decay $f(c) = (c+\delta_0)^{-\beta}$ in the far field ($c \gg \delta_0$), the relative interaction error is

$$\frac{f(\hat{c})}{f(c)} - 1 \;\approx\; (1+e)^{-\beta} - 1 \;\approx\; -\beta e + \tfrac{1}{2}\beta(\beta+1)\,e^2,$$

so its expectation over pairs is

$$\mathbb{E}\left[\frac{f(\hat{c})}{f(c)} - 1\right] \;\approx\; \tfrac{1}{2}\,\beta(\beta+1)\,\sigma^2 \;-\; \beta\mu. \tag{2}$$

The first term is the Jensen (convexity) contribution: it is positive even when the distance error is unbiased ($\mu = 0$) and grows quadratically in the distance spread and superlinearly in the kernel steepness β. The second term converts any systematic distance bias into interaction bias; empirically the hierarchical effective cost slightly underestimates exact distances ($\mu < 0$), which *adds* to the positive interaction bias.

On the GB driving network (main text, Section 6), the far-field relative distance error—computed over the 468,895 sampled pairs beyond the finest-layer cutoff $\alpha\rho_0 = 120\,\text{s}$—has $\sigma \approx 0.196$ and $\mu \approx -0.027$, so (2) predicts biases of +6.5% ($\beta = 1$), +11% ($\beta = 1.5$), and +17% ($\beta = 2$). The observed accessibility-level biases (+3.7%, +7.2%, and +14.9% / +16.5% respectively, SI Table S4) reproduce the predicted sign, ordering, and approximate magnitude; the prediction slightly overestimates because near-field pairs are exact and σ varies across bins. Equation (2) thus provides a practical a priori estimate of the systematic component of the operator's error, which can be calibrated away by rescaling with the total interaction mass ratio (SI Table S4, column ε_{cal}). This variance-driven correction is the graph-side analogue of the tolerance-driven truncation analyses used in continuous hierarchical methods, where admissibility conditions control the same second-order term.

Connection to $\mathcal{H}$-matrix admissibility

In the $\mathcal{H}$-matrix framework, a block (t, s) is *admissible* (i.e. compressible) if the clusters t and s are well-separated relative to their diameters: $\text{dist}(t,s) \geq \eta \cdot \min(\text{diam}(t), \text{diam}(s))$ for some $\eta > 0$. Our hierarchy enforces an analogous condition: at layer k, representative pairs are stored only within cutoff $\alpha \cdot \rho_k$, while group radii satisfy

$$\delta_k \;\leq\; \sum_{j=1}^{k} \frac{\rho_j}{2} \;=\; \frac{\gamma}{2(\gamma-1)}\left(1-\gamma^{-k}\right)\rho_k,$$

since a node's distance to its layer-k representative accumulates through at most k propagation steps, each bounded by $\rho_j/2$. Hence $\delta_k = O(\rho_k)$, staying within a constant factor of the layer radius: $\delta_k < \rho_k$ for the common choice $\gamma \geq 2$, and $\delta_k \leq \frac{\gamma}{2(\gamma-1)}\,\rho_k$ in general (the factor exceeds one only for $\gamma < 2$). The overlap factor α thus plays a role analogous to the admissibility parameter η: larger α increases the accuracy of coarse-layer approximations at the cost of additional computation, mirroring the accuracy–efficiency tradeoff in $\mathcal{H}$-matrix constructions.

Dynamic Update Analysis

Node and link additions

The hierarchy admits an incremental update framework in which update propagation is proportional to the number of affected pairs times the hierarchy depth. We outline this framework

here; the current public HierX release does not yet include dynamic updates, and a full empirical validation is deferred to future work.

Node addition. When a new node v is added, connected by a single link to an existing node u, the hierarchy must be updated to include v's costs, group assignments, and interaction entries at each layer. Let n_{loc} denote the number of nodes reachable from v within the finest-layer cutoff $\alpha \cdot \rho_0$. Because v enters the network through u, the cost from v to any node w is simply $c_{vw} = c_{vu} + c_{uw}$; since u's costs to nearby nodes are already stored in the hierarchy, the required costs for v can be obtained by a simple offset rather than by running new shortest-path searches. With an incremental correction scheme (updating only entries involving v), the total work is

$$T_{\text{add node}} \approx O(n_{\text{loc}} \cdot K).$$

In typical spatial networks n_{loc} is small, so node addition is effectively a local operation whose work does not grow with n.

Link addition. Adding a link between existing nodes p and q can improve shortest-path costs c_{ij} for many node pairs. Every improved path must use the new link, so the affected pairs are exactly those (i, j) for which $c_{ip} + c_{pq} + c_{qj} < c_{ij}$ (or the reverse). Two cutoff-limited Dijkstra runs—one from p, one from q, each limited to $\alpha \cdot \rho_K$—suffice to identify all such pairs. Let n_p and n_q denote the number of nodes reached by these two runs; the number of affected pairs is then $\Delta \leq n_p \cdot n_q$.

The new costs $c_{ip} + c_{pq} + c_{qj}$ are already available from the same two runs, so no additional shortest-path computation is needed. For each of the Δ improved pairs, the stored costs and interactions are updated and the change is propagated along the chain of ancestor groups. Each node appears in $O(K)$ groups, so propagation depth is $O(K)$. The total update work is therefore

$$T_{\text{add link}} = \underbrace{O(n_p \log n_p + n_q \log n_q)}_{\text{two Dijkstra runs}} + \underbrace{O(\Delta \cdot K)}_{\text{operator update}} .$$

In spatial networks a new link typically affects only a limited region, so n_p, n_q, and Δ are small relative to n. The propagation depth is logarithmic in n, and total work scales with the size of the impact region rather than with overall network size.

Additional Discussion

Directed graphs and asymmetric costs. Real transport networks often include directional links (one-way streets, transit routes). The operator formulation generalizes straightforwardly to directed graphs: the interaction matrices F_k become asymmetric, and the correction logic remains valid because it depends only on the existence of stored entries, not on symmetry. However, the distance discrepancy bound (Eq. 7 in the main text) must be adapted: on directed graphs, the group radius δ_k must account for both forward and backward distances between nodes and their representatives ($\delta_k = \max_i \max(c(i, r_k[i]),\, c(r_k[i], i))$), since the directed triangle inequality $c(i,j) \leq c(i,r) + c(r,s) + c(s,j)$ involves directional paths. The per-entry bound $2\, L_f\, \delta_k$ remains valid under this redefinition. In practice, transport networks have mild asymmetry (most links are bidirectional), so the error increase is expected to be modest.

Parallelization. The hierarchical operator is naturally suited to parallel computation. At each layer, the Dijkstra searches from different representatives are independent and can be executed concurrently. The layer-wise matrix–vector products $(F_k - \text{Corr}_k)\, \mathbf{x}_k$ are likewise independent across layers and can be computed in parallel. With $K = O(\log N)$ layers and $O(n/K)$ work per layer, the parallel depth of a single operator application is $O(\log n)$ with $O(n)$ total

work—matching the theoretical parallel complexity of fast multipole methods. In our Python implementation, the build phase dominates runtime and benefits substantially from multi-core execution of the Dijkstra searches.

London Baseline: Moderate and Exponential Kernels

On the 61,432-node London driving network (13 km radius, mean edge cost $\bar{c} = 9.1$ s, network diameter 3,181 s $\approx$ 53 min), we report detailed per-kernel results for the moderate power law $(c + 600)^{-1}$ and exponential $e^{-c/600}$ kernels, complementing the steep and very steep kernels discussed in the main text.

Measured by intrinsic Dijkstra work, the hierarchical operator offers the best accuracy-to-work ratio on steep kernels. On broader kernels, Nyström dominates the error-work tradeoff: even $m = 100$ landmarks (6.1 M nodes explored) achieve lower error than the hierarchical operator at $\alpha = 2$ (15.8 M nodes) on both the moderate and exponential kernels (SI Fig. S5). However, on the very steep kernel $(c + 60)^{-2}$, the Nyström approximation fails: even $m = 1{,}000$ landmarks yield 13.5% RMSE, while the hierarchical operator at $\alpha = 2$ already achieves 8.6% RMSE with $\sim$4$\times$ less work.
